# Opportunities and Challenges in Applying AI to Evolutionary Morphology

**DOI:** 10.1093/iob/obae036

**Published:** 2024-09-23

**Authors:** Y He, J M Mulqueeney, E C Watt, A Salili-James, N S Barber, M Camaiti, E S E Hunt, O Kippax-Chui, A Knapp, A Lanzetti, G Rangel-de Lázaro, J K McMinn, J Minus, A V Mohan, L E Roberts, D Adhami, E Grisan, Q Gu, V Herridge, S T S Poon, T West, A Goswami

**Affiliations:** L ife Sciences, Natural History Museum, London, UK; L ife Sciences, Natural History Museum, London, UK; Department of Ocean & Earth Science, National Oceanography Centre Southampton, University of Southampton, Southampton, UK; L ife Sciences, Natural History Museum, London, UK; Division of Biosciences, University College London, London, UK; AI and Innovation, Natural History Museum, London, UK; Digital, Data and Informatics, Natural History Museum, London, UK; L ife Sciences, Natural History Museum, London, UK; Department of Anthropology, University College London, London, UK; L ife Sciences, Natural History Museum, London, UK; L ife Sciences, Natural History Museum, London, UK; Department of Life Sciences, Imperial College London, London, UK; Grantham Institute, Imperial College London, London, UK; L ife Sciences, Natural History Museum, London, UK; Grantham Institute, Imperial College London, London, UK; Department of Earth Science and Engineering, Imperial College London, London, UK; L ife Sciences, Natural History Museum, London, UK; Centre for Integrative Anatomy, University College London, London, UK; L ife Sciences, Natural History Museum, London, UK; School of Geography, Earth and Environmental Sciences, University of Birmingham, Birmingham, UK; L ife Sciences, Natural History Museum, London, UK; School of Oriental and African Studies, London, UK; L ife Sciences, Natural History Museum, London, UK; Department of Earth Sciences, University of Oxford, Oxford, UK; L ife Sciences, Natural History Museum, London, UK; School of Biological and Behavioural Sciences, Queen Mary University of London, London, UK; L ife Sciences, Natural History Museum, London, UK; Biodiversity Genomics Laboratory, Institute of Biology, University of Neuchâtel, Neuchâtel, Switzerland; L ife Sciences, Natural History Museum, London, UK; L ife Sciences, Natural History Museum, London, UK; Department of Life Sciences, Imperial College London, London, UK; Imaging and Analysis Centre, Natural History Museum, London, UK; School of Engineering, London South Bank University, London, UK; AI and Innovation, Natural History Museum, London, UK; Digital, Data and Informatics, Natural History Museum, London, UK; L ife Sciences, Natural History Museum, London, UK; School of Biosciences, University of Sheffield, Sheffield, UK; AI and Innovation, Natural History Museum, London, UK; Digital, Data and Informatics, Natural History Museum, London, UK; Centre for Integrative Anatomy, University College London, London, UK; Imaging and Analysis Centre, Natural History Museum, London, UK; L ife Sciences, Natural History Museum, London, UK

## Abstract

Artificial intelligence (AI) is poised to revolutionize many aspects of science, including the study of evolutionary morphology. While classical AI methods such as principal component analysis and cluster analysis have been commonplace in the study of evolutionary morphology for decades, recent years have seen increasing application of deep learning to ecology and evolutionary biology. As digitized specimen databases become increasingly prevalent and openly available, AI is offering vast new potential to circumvent long-standing barriers to rapid, big data analysis of phenotypes. Here, we review the current state of AI methods available for the study of evolutionary morphology, which are most developed in the area of data acquisition and processing. We introduce the main available AI techniques, categorizing them into 3 stages based on their order of appearance: (1) machine learning, (2) deep learning, and (3) the most recent advancements in large-scale models and multimodal learning. Next, we present case studies of existing approaches using AI for evolutionary morphology, including image capture and segmentation, feature recognition, morphometrics, and phylogenetics. We then discuss the prospectus for near-term advances in specific areas of inquiry within this field, including the potential of new AI methods that have not yet been applied to the study of morphological evolution. In particular, we note key areas where AI remains underutilized and could be used to enhance studies of evolutionary morphology. This combination of current methods and potential developments has the capacity to transform the evolutionary analysis of the organismal phenotype into evolutionary phenomics, leading to an era of “big data” that aligns the study of phenotypes with genomics and other areas of bioinformatics.

## Synopsis (Polish)

Sztuczna inteligencja (AI) może w przyszłości zrewolucjonizować wiele aspektów nauki, w tym badanie morfologii ewolucyjnej. Chociaż klasyczne instrumenty sztucznej inteligencji, takie jak analiza głównych składowych i analiza skupień, są powszechne od dziesięcioleci w badaniach morfologii ewolucyjnej, w ostatnich latach obserwuje się coraz szersze zastosowanie uczenia głębokiego (depp learning) w ekologii i biologii ewolucyjnej. W miarę jak cyfrowe bazy danych okazów stają się coraz bardziej powszechne i ogólnodostępne, sztuczna inteligencja oferuje nowy, ogromny potencjał w zakresie omijania długotrwałych barier utrudniających szybką analizę dużych zbiorów danych fenotypowych. Prezentujemy przegląd obecnego stanu wiedzy o najbardziej rozwiniętych metodach AI używanych w badaniach morfologii ewolucyjnej do pozyskiwania i przetwarzania danych. Przedstawiamy główne dostępne techniki sztucznej inteligencji, dzieląc je na trzy etapy w zależności od kolejności ich występowania: (1) uczenie maszynowe, (2) uczenie głębokie oraz (3) najnowsze osiągnięcia w modelach wielkoskalowych i uczeniu multimodalnym. Następnie przedstawiamy studium przypadków wykorzystujących sztuczną inteligencję w badaniach morfologii ewolucyjnej, w tym przechwytywania i segmentacji obrazu, rozpoznawania cech, morfometrii i filogenetyki. Następnie omawiamy perspektywę krótkoterminowych postępów w konkretnych obszarach badań w tej dziedzinie, w tym potencjał nowych metod sztucznej inteligencji, które nie znalazły jeszcze zastosowania w badaniach nad morfologią ewolucyjną. W szczególności zwracamy uwagę na kluczowe obszary, w których sztuczna inteligencja pozostaje jeszcze niewykorzystana i można ją wykorzystać do usprawnienia badań nad morfologią ewolucyjną. To połączenie obecnych metod i potencjalnych rozwiązań może w przyłości przekształcić analizę ewolucyjną fenotypu organizmu w fenomenologię ewolucyjną, prowadząc do ery „dużych zbiorów danych", które dopasowują badanie fenotypów do genomiki i innych dziedzin bioinformatyki.

## Synopsis (Simplified Chinese)

人工智能正处于变革科学诸多领域的前沿, 进化形态学也不例外。尽管诸如主成分分析和聚类分析等传统方法已经在进化形态学研究中应用数十年, 但近年来, 深度学习在生态学和进化生物学中的应用日益增多。随着数字化标本数据库的广泛普及和开放共享, 人工智能展现出巨大的潜力, 能够克服长期以来高速分析大量表型数据中的效率障碍。在此, 我们回顾了当前用于进化形态学研究的人工智能方法, 发现这些方法的应用主要集中在数据获取和处理方面。本文中, 我们先介绍了现有的主要人工智能技术, 并根据它们出现的顺序将其分类为三个阶段:(1)机器学习, (2)深度学习, 和(3)最近的大规模模型和多模态学习。接下来, 我们通过案例分析展示了利用人工智能进行进化形态学研究的现有方法, 包括图像捕捉与分割、特征识别、形态计量学和系统发育学。随后, 我们探讨了该领域在特定研究方向上的近期进展前景, 特别是尚未应用于形态进化研究的潜在人工智能方法。此外, 我们还指出了人工智能在若干关键领域中应用的不足之处, 并探讨了其在进一步提升进化形态学研究中的潜在价值。这种当前方法与未来发展的结合, 能够将表型的进化分析转变为进化表型学。使得在大数据时代下, 表型研究与基因组学及其他生物信息学领域接轨。

## Synopsis (Traditional Chinese)

人工智慧正處於變革科學諸多領域的前沿, 進化形態學也不例外。儘管主成分分析和群聚分析等傳統方法已在進化形態學研究中應用數十年, 但近年來, 深度學習在生態學和進化生物學中的應用日益增加。隨著數位化標本資料庫的廣泛普及與開放共享, 人工智慧展現出巨大的潛力, 能夠克服長期以來高速分析大量表型資料中的效率障礙。本文回顧了目前用於進化形態學研究的人工智慧方法, 並發現這些方法的應用主要集中在資料獲取與處理方面。本文中, 我們先介紹現有的主要人工智慧技術, 並依據它們出現的順序將其分為三個階段:(1)機器學習, (2)深度學習, 以及(3)最近的大規模模型和多模態學習。接下來, 我們透過案例分析展示了利用人工智慧進行進化形態學研究的現有方法, 包括影像擷取與分割、特徵識別、形態計量學與系統發育學。隨後, 我們探討了該領域在特定研究方向上的近期進展前景, 特別是尚未應用於形態進化研究的潛在人工智慧方法。此外, 我們還指出了人工智慧在若干關鍵領域中應用的不足之處, 並探討了其在進一步提升進化形態學研究中的潛在價值。當前方法與未來發展的結合, 有望將表型的進化分析轉變為進化表型學, 使表型研究在大數據時代能與基因體學及其他生物資訊學領域接軌。

## Synopsis (Japanese)

人工知能(AI)は、進化形態学を含む多くの科学分野において、革新をもたらす大きな可能性を秘めています。主成分分析やクラスター分析といった従来の手法は、何十年にもわたって進化形態学の研究に用いられてきましたが、近年では深層学習の応用が生態学および進化生物学において急速に進展しています。デジタル化された標本データベースがますます普及し、公開される中で、AIはこれまでの制約を打破し、迅速かつ大規模な表現型データの解析を可能にする大きな潜在力を示しています。 本論文では、現在進化形態学の研究に利用されているAI技術の現状を概観し、その多くがデータ取得および処理に集中していることを明らかにします。さらに、技術の発展過程に基づいて、AI技術を以下の3つの段階に分類します:(1)機械学習、(2)深層学習、(3)最新の大規模モデルおよびマルチモーダル学習。次に、AIを活用した進化形態学研究の実例として、画像追跡と分割、特徴認識、形態計量学、系統学の事例を紹介します。 続いて、形態進化の研究において、まだ十分に応用されていない新たなAI技術の可能性について議論し、今後の具体的な研究分野における短期的な進展の見通しを探ります。特に、AIが十分に活用されていない重要な分野を指摘し、それが進化形態学研究のさらなる発展にどのように寄与し得るかを検討します。このように、現行の方法と今後の技術進歩が融合することで、表現型の進化分析が進化表現型学へと発展し、ビッグデータ時代における表現型研究がゲノミクスや他の生命情報科学分野とより密接に統合されることが期待されます。

## Synopsis (Italian)

L'intelligenza artificiale (IA) è destinata a rivoluzionare molti aspetti della scienza, incluso lo studio della morfologia evolutiva. Mentre metodi classici di IA, come l'analisi delle componenti principali e l'analisi dei cluster, sono stati comunemente utilizzati nello studio della morfologia evolutiva per decenni, negli ultimi anni si è assistito a un aumento dell'applicazione del deep learning all'ecologia e alla biologia evolutiva. Con la crescente diffusione e disponibilità di database aperti di esemplari digitalizzati, l'IA offre nuove potenzialità per superare le barriere che storicamente hanno impedito l'analisi rapida di grandi quantità di dati di fenotipi. In questo lavoro, esaminiamo lo stato attuale dei metodi di IA disponibili per lo studio della morfologia evolutiva, che si sono per lo più sviluppati nell'acquisizione e nella lavorazione dei dati. Forniamo un'introduzione alle principali tecniche di IA disponibili, suddividendole in tre tipi in base all'ordine in cui sono state utilizzate per la prima volta in studi: (1) machine learning, (2) deep learning e (3) gli sviluppi più recenti nei modelli su larga scala e nel multimodal learning. Successivamente, presentiamo esempi di studi che utilizzano metodi esistenti di IA in diverse applicazioni nel campo della morfologia evolutiva, inclusi la cattura e segmentazione delle immagini, il riconoscimento dei caratteri, la morfometria e la filogenetica. Discutiamo poi di avanzamenti futuri nel breve periodo in aree di ricerca specifiche all'interno di questo campo, incluse potenziali applicazioni di metodi di IA che non sono ancora stati applicati allo studio della morfologia evolutiva. In particolare, evidenziamo alcune aree importanti in cui l'IA è ancora sotto-utilizzata e potrebbe essere impiegata per migliorare gli studi di morfologia evolutiva. I metodi attualmente utilizzati e i potenziali sviluppi hanno la capacità in modo combinato di trasformare le analisi sull'evoluzione del fenotipo degli organismi in “fenomica evolutiva”, una area di ricerca innovata che grazie ai “big data” allinea lo studio dei fenotipi con la genomica e altre aree della bioinformatica.

## Synopsis (French)

L'intelligence artificielle (IA) est destinée à révolutionner de nombreux aspects de la science, y compris l'étude de la morphologie évolutive. Alors que les méthodes classiques d'IA, telles que l'analyse en composantes principales et l'analyse de clusters, ont été couramment utilisées dans l'étude de la morphologie évolutive depuis des décennies, ces dernières années ont vu une augmentation de l'application du deep learning à l'écologie et à la biologie évolutive. Avec la diffusion croissante et la disponibilité de bases de données ouvertes d'échantillons numérisés, l'IA offre de nouvelles potentialités pour surmonter les barrières qui ont historiquement empêché l'analyse rapide de grandes quantités de données sur les phénotypes. Dans ce travail, nous examinons l'état actuel des méthodes d'IA disponibles pour l'étude de la morphologie évolutive, qui se sont principalement développées dans l'acquisition et le traitement des données. Nous fournissons une introduction aux principales techniques d'IA disponibles, en les divisant en trois types en fonction de l'ordre dans lequel elles ont été utilisées pour la première fois dans les études: (1) l'apprentissage automatique (machine learning), (2) l'apprentissage profond (deep learning) et (3) les développements les plus récents dans les modèles à grande échelle et l'apprentissage multimodal (multimodal learning). Ensuite, nous présentons des exemples d'études utilisant des méthodes d'IA existantes dans diverses applications dans le domaine de la morphologie évolutive, y compris la capture et la segmentation d'images, la reconnaissance des caractères, la morphométrie et la phylogénétique. Nous discutons ensuite des avancées futures à court terme dans des domaines de recherche spécifiques de ce champ, y compris des applications potentielles de méthodes d'IA qui n'ont pas encore été appliquées à l'étude de la morphologie évolutive. En particulier, nous mettons en évidence certaines zones importantes où l'IA est encore sous-utilisée et pourrait être utilisée pour améliorer les études de morphologie évolutive. Les méthodes actuellement utilisées et les développements potentiels ont la capacité combinée de transformer l'analyse de l'évolution des phénotypes des organismes en “phénomique évolutive”, un domaine de recherche innovant qui, grâce aux “big data”, aligne l'étude des phénotypes avec la génomique et d'autres domaines de la bio-informatique.

## Synopsis (German)

Künstliche Intelligenz (KI) hat tiefgreifende Änderungen in vielen Aspekten der Wissenschaft ausgelöst, so auch im Forschungsbereich der evolutionären Morphologie. Während klassische KI-Methoden wie die Hauptkomponentenanalyse und die Clusteranalyse [OH1] bei der Untersuchung der evolutionären Morphologie seit Jahrzehnten gang und gäbe sind, wurde in den letzten Jahren zunehmend Deep Learning in der Ökologie und Evolutionsbiologie eingesetzt. Da eine wachsende Masse von Forschungsdaten in digitalen Datenbanken öffentlich zugänglich gemacht wird, können mittels KI nun die Überwindung schnelle und gleichzeitig umfangreiche phänotyopische Analysen basierend auf big data durchgeführt werden. In dieser Arbeit geben wir einen Überblick über den aktuellen Stand der für die Untersuchung der evolutionären Morphologie verfügbaren und im Bereich der Datenerfassung und -verarbeitung am weitesten entwickelten KI-Methoden. Wir stellen die wichtigsten verfügbaren KI-Techniken vor und drei nacheinander aufgetretenen Stufen zu: (1) maschinelles Lernen, (2) Deep Learning und (3) die jüngsten Fortschritte bei groß angelegten Modellen und multimodalem Lernen. Danach stellen wir Fallstudien zu bestehenden Ansätzen der Nutzung von KI im Bereich der evolutionären Morphologie vor, beispielsweise Bilderfassung und Segmentierung, Merkmalserkennung, Morphometrie und Phylogenetik. Anschließend diskutieren wir mögliche Fortschritte in bestimmten Bereichen der Forschung auf diesem Gebiet in naher Zukunft, darunter auch solche KI-Methoden, die bisher noch nicht zur Untersuchung der morphologischen Evolution angewendet wurden. Wir weisen insbesondere auf Schlüsselbereiche hin, in denen noch weiteres Potenzial zur Nutzung von KI besteht und die zur Verbesserung der Studien zur evolutionären Morphologie eingesetzt werden könnten. Diese Kombination aus bestehenden Methoden und aktuellen Neuentwicklungen könnte die evolutionäre Analyse des Phänotyps von Organismen in eine evolutionäre Phänomik verwandeln, die in enger Verbindung mit der Genomik und anderen Bereichen der Bioinformatik zu einer Ära von integrativer „big data“ führt.

## Synopsis (Malagasy)

Ny faharanitan-tsaina artifisialy (artificial intelligence—AI) dia afaka ny hampiova sy hanavao ny lafiny maro eo amin'ny siansa, anisan'izany ny fandalinana ny morfolojia evolisionera (evolutionary morphology). Na dia efa nampiasaina nandritra ny taona maro tamin'ny morfolojia evolisionera aza ny fomba AI klasika toy ny fanadihadiana ny singa fototra (principal component analysis) sy ny fanasokajiana (Cluster Analysis), dia hita fa mihamaro ny fampiasana ny fianarana lalina (deep learning) eo amin'ny ekolojia sy ny biolojia evolisionera tato anatin'ny taona vitsivitsy. Efa mihamaro sy misokatra amin'ny besinimaro ireo tahiry elektronika misy ireo santionany ankehitriny, ka dia manome fahafahana goavana ny AI hanampy amin'ny famahana ny sakana lehibe amin'ny fanadihadiana haingana ireo tahiry goavana momba ny fenôtypika. Ato amin'ity asa ity, dia jerentsika ny toeran'ny fandrosoana ankehitriny amin'ny fampiasana AI amin'ny fandalinana ny morfolojia evolisionera, izay efa nandroso indrindra eo amin'ny sehatry ny fakana sy fanodinana angona. Asehontsika ireo teknika AI lehibe azo ampiasaina, izay mizara telo arakaraka ny filaharany amin'ny vanim-potoana nipoirany: (1) ny fianarana milina (machine learning), (2) ny fianarana lalina (deep learning), ary (3) ny fandrosoana farany indrindra amin'ny modely lehibe sy ny fianarana mitambatra maromaro (multimodal learning). Avy eo dia asehontsika ireo tranga efa nampiasana AI amin'ny morfolojia evolisionera, anisan'izany ny fakana sy fizarana sary, ny fahafantarana ny endri-javatra, ny morfometrika, ary ny fiaviana (phylogenetics). Manaraka izany, dia resahintsika ny fanantenana ho amin'ny fivoarana akaiky amin'ny lafiny manokana eo amin'ity sehatra ity, anisan'izany ny fampiasana ireo fomba AI vaovao izay mbola tsy nampiasaina amin'ny fandalinana ny fivoaran'ny morfolojia. Asongadintsika manokana ireo tontolo lehibe izay mbola tsy ampy fampiasana AI ary azo ampiasaina hanatsarana ny fandalinana ny morfolojia evolisionera. Ity fampitambarana ireo fomba ankehitriny sy ny fandrosoana mety hitranga ity dia manana ny fahefana manova tanteraka ny fandalinana evolisionera ny fenôtypika ho lasa “big data” ka mampifanakaiky ny fandalinana ny fenôtypika sy ny genomika ary ny sehatra hafa ao amin'ny bioinformatika.

## Synopsis (Spanish)

La inteligencia artificial (IA) está destinada a revolucionar muchos aspectos de la ciencia, incluido el estudio de la morfología evolutiva. Aunque los métodos clásicos de IA, como el análisis de componentes principales y el análisis de clústeres, han sido habituales en el estudio de la morfología evolutiva durante décadas, en los últimos años se ha observado una creciente aplicación del aprendizaje profundo a la ecología y la biología evolutiva. A medida que las bases de datos de especímenes digitalizados se vuelven cada vez más frecuentes y están disponibles de forma abierta, la IA está ofreciendo un nuevo y vasto potencial para sortear las barreras existentes desde hace mucho tiempo para el análisis rápido de grandes datos de fenotipos. Aquí revisamos el estado actual de los métodos de IA disponibles para el estudio de la morfología evolutiva, que están más desarrollados en el área de adquisición y procesamiento de datos. Presentamos las principales técnicas de IA disponibles, categorizándolas en tres etapas en función de su orden de aparición: (1) aprendizaje automático, (2) aprendizaje profundo, y (3) los avances más recientes en modelos a gran escala y aprendizaje multimodal. A continuación, presentamos estudios de casos de enfoques existentes que utilizan la IA para la morfología evolutiva, incluyendo la captura y segmentación de imágenes, el reconocimiento de rasgos, la morfometría y la filogenética. A continuación, analizamos las perspectivas de avances a corto plazo en áreas específicas de investigación dentro de este campo, incluido el potencial de nuevos métodos de IA que aún no se han aplicado al estudio de la evolución morfológica. En concreto, señalamos las áreas clave en las que la IA sigue infrautilizada y que podrían utilizarse para mejorar los estudios de morfología evolutiva. Esta combinación de métodos actuales y desarrollos potenciales tiene la capacidad de transformar el análisis evolutivo del fenotipo del organismo en fenómica evolutiva, conduciendo a una era de “big data“ que alinee el estudio de los fenotipos con la genómica y otras áreas de la bioinformática.

## Synopsis (Catalan)

La intel·ligència artificial (IA) està destinada a revolucionar molts aspectes de la ciència, inclòs l'estudi de la morfologia evolutiva. Encara que els mètodes clàssics de IA, com l'anàlisi de components principals i l'anàlisi de clústers, han estat habituals en l'estudi de la morfologia evolutiva durant dècades, en els últims anys s'ha observat una creixent aplicació de l'aprenentatge profund a l'ecologia i la biologia evolutiva. A mesura que les bases de dades d'espècimens digitalitzats es tornen cada vegada més freqüents i estan disponibles de manera oberta, la IA està oferint un nou i vast potencial per a eludir les barreres existents des de fa molt temps per a l'anàlisi ràpida de grans dades de fenotips. Aquí revisem l'estat actual dels mètodes de IA disponibles per a l'estudi de la morfologia evolutiva, que estan més desenvolupats en l'àrea d'adquisició i processament de dades. Presentem les principals tècniques de IA disponibles, categoritzant-les en tres etapes en funció de la seva ordre d'aparició: (1) aprenentatge automàtic, (2) aprenentatge profund, i (3) els avanços més recents en models a gran escala i aprenentatge multimodal. A continuació, presentem estudis de casos d'enfocaments existents que utilitzen la IA per a la morfologia evolutiva, incloent-hi la captura i segmentació d'imatges, el reconeixement de trets, la morfometria i la filogenètica. A continuació, analitzem les perspectives d'avanços a curt termini en àrees específiques de recerca dins d'aquest camp, inclòs el potencial de nous mètodes de IA que encara no s'han aplicat a l'estudi de l'evolució morfològica. En concret, assenyalem les àrees clau en les quals la IA segueix infrautilitzada i que podrien utilitzar-se per a millorar els estudis de morfologia evolutiva. Aquesta combinació de mètodes actuals i desenvolupaments potencials té la capacitat de transformar l'anàlisi evolutiva del fenotip de l'organisme en fenómica evolutiva, conduint a una era de “big data“ que alineï l'estudi dels fenotips amb la genòmica i altres àrees de la bioinformàtica.

## Synopsis (Norwegian)

Kunstig intelligens (KI) er i ferd med å revolusjonere mange aspekter av vitenskap, inkludert studiet av evolusjonær morfologi. Selv om klassiske KI-metoder som hovedkomponentanalyse og klyngeanalyse har vært vanlige i studiet av evolusjonær morfologi i flere tiår, har de senere årene sett økende bruk av dyp læring innen økologi og evolusjonsbiologi. Etter hvert som digitaliserte eksemplardatabaser blir stadig mer utbredt og åpent tilgjengelige, tilbyr KI et stort potensial for å omgå langvarige hindringer for rask, storskala dataanalyse av fenotyper. Her gjennomgår vi dagens KI-metoder tilgjengelig for studiet av evolusjonær morfologi, som er mest utviklet innen dataregistrering og prosessering. Vi introduserer de viktigste tilgjengelige KI-teknikkene, og kategoriserer dem i tre stadier basert på deres rekkefølge av fremvekst: (1) maskinlæring, (2) dyp læring, og (3) de nyeste fremskrittene innen storskalamodeller og multimodal læring. Deretter presenterer vi casestudier av eksisterende tilnærminger som bruker KI for evolusjonær morfologi, inkludert bildeinnsamling og segmentering, funksjonsgjenkjenning, morfometri og fylogenetikk. Vi diskuterer deretter fremtidige fremskritt på spesifikke forskningsområder innen dette feltet, inkludert potensialet til nye KI-metoder som ennå ikke er anvendt på studiet av morfologisk evolusjon. Spesielt påpeker vi nøkkelområder hvor KI fortsatt er underutnyttet og kan brukes til å forbedre studier av evolusjonær morfologi. Denne kombinasjonen av nåværende metoder og potensielle utviklinger har kapasitet til å forvandle den evolusjonære analysen av organismens fenotype til evolusjonær phenomics, og innlede en æra med “big data” som bringer studiet av fenotyper i tråd med genomics og andre områder av bioinformatikk.

## Synopsis (Portuguese)

A inteligência artificial (IA) está preparada para revolucionar muitos aspectos da ciência, incluindo o estudo da morfologia evolutiva. Embora os métodos clássicos de IA, como a análise de componentes principais e a análise de agrupamentos, tenham sido comuns no estudo da morfologia evolutiva durante décadas, nos últimos anos temos visto uma aplicação crescente da aprendizagem profunda à ecologia e à biologia evolutiva. À medida que os bancos de dados de espécimes digitalizados se tornam cada vez mais predominantes e disponíveis abertamente, a IA oferece um novo e vasto potencial para contornar barreiras de longa data à análise rápida de fenótipos de big data. Aqui, revisamos o estado atual dos métodos de IA disponíveis para o estudo da morfologia evolutiva, que são mais desenvolvidos na área de aquisição e processamento de dados. Apresentamos as principais técnicas de IA disponíveis, categorizando-as em três estágios com base em sua ordem de aparecimento: (1) o ‘machine learning’, (2) o ‘deep learning’ e (3) os avanços mais recentes em modelos de grande escala e o ‘multimodal learning’. A seguir, apresentamos estudos de caso de abordagens existentes usando IA para morfologia evolutiva, incluindo captura e segmentação de imagens, reconhecimento de características, morfometria e filogenética. Em seguida, discutimos o prospecto para avanços de curto prazo em áreas específicas de investigação neste campo, incluindo o potencial de novos métodos de IA que ainda não foram aplicados ao estudo da evolução morfológica. Em particular, notamos áreas-chave onde a IA permanece subutilizada e poderia ser usada para aprimorar estudos de morfologia evolutiva. Esta combinação de métodos atuais e desenvolvimentos potenciais tem a capacidade de transformar a análise evolutiva do fenótipo do organismo em fenômica evolutiva, levando a uma era de “big data” que alinha o estudo dos fenótipos com a genômica e outras áreas da bioinformática.

## Synopsis (Dutch)

Artificiële intelligentie (AI) staat paraat om revolutionair te zijn voor verschillende aspecten van de wetenschap, inclusief de studie van evolutionaire morfologie. Het gebruik van klassieke AI-methodes zoals principal component analyse and cluster analyse wordt al decennia lang regelmatig gebruikt in de studie van evolutionaire morfologie, maar in recente jaren is het gebruik van “deep learning” in de studie van ecologie en evolutionaire biologie toegenomen. Nu dat het digitaliseren van exemplaar databases meer regelmatig en vaak openbaar is, offert AI veel nieuwe mogelijkheden om lang bestaande barrières te omringen, inclusief het spoedige analyse van “big data” van fenotypen. In deze wetenschappelijke beoordeling vatten we de beschikbare AI-modellen samen die geschikt staan voor de study van evolutionaire morfologie met de focus op diegene die het meest geschikt staan voor data-acquisitie en verwerking. Wij introduceren de voornaamste AI-technieken en sorteren ze in drie groepen gebaseerd op de volgorde van verschijning: (1) machinelearning, (2) deep learning, en (3) de meest recente vooruitgang in vorderingen van grootschalige modellen en multimodaal leren. Daarna presenteren wij casestudies van de bestaande gebruiksmiddelen van AI voor de wetenschap van evolutionaire morfologie, inclusief het gebruik voor beeld vastleggen en segmentatie, kenmerken herkenning, morfometrie en fylogenetica. We besprekenook hetvooruitzicht van de vooruitgang op korte termijn op dit gebied, inclusief de potentie van nieuwe AI-methodes die nog niet gebruikt worden binnen het gebied van evolutionaire morfologie. We bespreken vooral de gebieden waar AI onderbenut wordt en waar het gebruik van AI de studie van evolutionaire morfologie versterkt. De combinatie van huidige methoden en de potentiële ontwikkelingen daarvan hebben de capaciteit om de evolutionaire analyse van het organismale fenotype naar evolutionaire fenomica te transformeren, leidend tot “big data'' dat de gebieden van fenotypen en genomica en andere gebieden van de bio-informatica samen brengt.

## Synopsis (Papiamento)

Inteligensia Artificial (IA) ta para cla pa revolutioná siensa den hopi sentido, incluyendo den e studio di morfologia evolucionario. E uso di metodonan classico di IA manera análisis di componente principal y análisis di cluster, den e estudio di morfologia evolucionario ta comun pa década. Den e ultimo anjanan tin un cresemento di e application di “deep learning” den e studio di ecologia y biologia evolucionario. Awor cu mas datonan ta wordo colecciona ambos digital y publico, IA ta ofrece un manara nobo pa evitá bareranan cu ta prevení e análisis lihe di “big data” relationando cu e estudio di fenotiponan. Den e revista científica aki nos ta declará e formanan di IA metodonan disponible pa e estudio di morfologia evolucionario cu ta mas desaroyá den e área di acuerdo y tratamento di data. Nos lo introduci e maneranan principal di IA y categorisá nan den: (1) machine learning, (2) deep learning, y (3) e desaroyonan mas reciente den “large-scale models” y “multimodal learning”. Tambe nos lo papia di estudio di metodonan ecsistente cu por wordo usa den morfologia evolucionario, incluyendo esnan uza pa e capturation y segmentacion di imagen, reconosemento di característica, morfometria y filogenetica. E or' ei nos ta discuti e potencial di metodonan nobo di IA cu te awor no a wordo usa den e studio di morfologia evolucionario. Particularmente, nos ta discuti e areanan unda IA no ta wordo utilizá optimalmente pa mehora e studio di morfologia evolucionario. E combinacion di e metodonan classico y e desaroyo di metodonan nobo tin e poder pa por transformá con nos ta analysá e fenotipo di organismo den fenomenologia evolucionario. Esaki lo resulta den un era di “big data” cu ta alinea cu e estudio di fenotiponan cu genomica y ortro areanan di bioinformática.

## Abstract (kn)

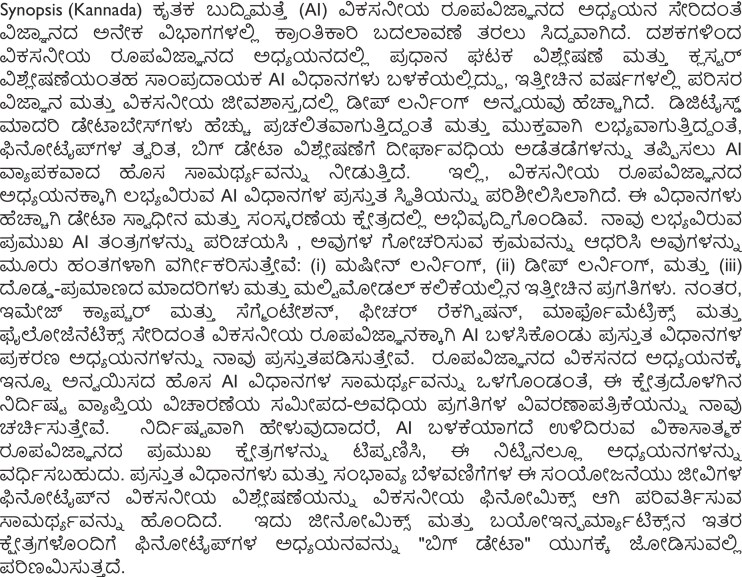

## Abstract (hi)

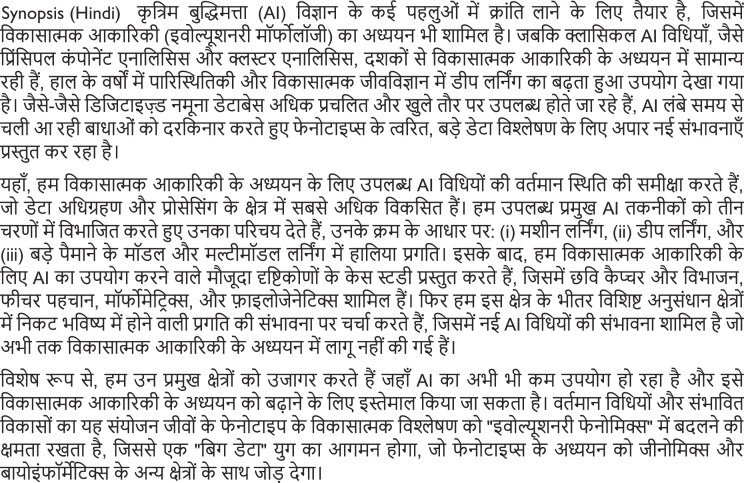

## Abstract (el)

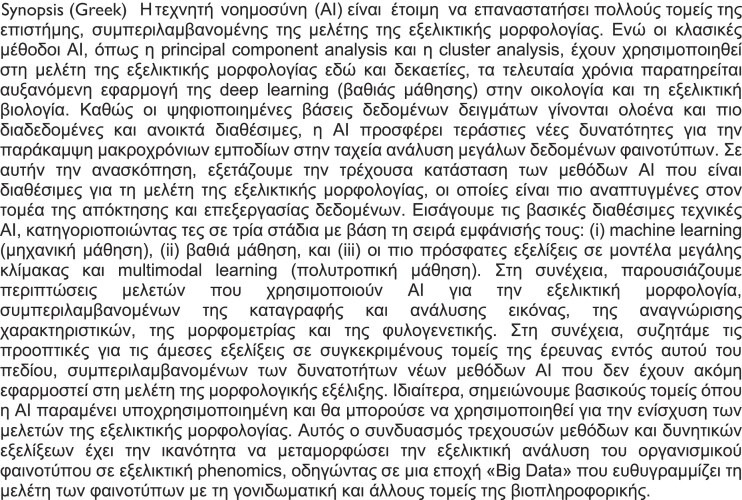

## Synopsis (Afrikaans)

Kunsmatige intelligensie (KI) is het die potensiaal om baie aspekte van die wetenskap te revolusioneer, insluitend die studie van evolusionêre morfologie. Terwyl klassieke KI-metodes soos hoofkomponent-analise en klusteranalise al dekades algemeen in die studie van evolusionêre morfologie is, het die afgelope jare toenemende toepassing van “deep learning” op ekologie en evolusionêre biologie gesien. Namate gedigitaliseerde monsterdatabasisse al hoe meer algemeen en openlik beskikbaar word, bied KI nuwe potensiaal om langdurige hindernisse vir vinnige, grootdata-ontleding van fenotipes te omseil. Hier hersien ons die huidige stand van KI-metodes wat beskikbaar is vir die studie van evolusionêre morfologie wat die meeste ontwikkel is op die gebied van data-verkryging en -verwerking. Ons stel die belangrikste beskikbare KI-tegnieke bekend en kategoriseer hulle in drie stadiums gebaseer op hul voorkomsvolgorde: (1) masjienleer, (2) “deep learning”, en (3) die mees onlangse vordering in grootskaalse modelle en multimodale leer. Volgende bied ons gevallestudies aan van bestaande benaderings wat KI gebruik vir evolusionêre morfologie, insluitend beeldvaslegging en segmentering, kenmerkherkenning, morfometrie en filogenetika. Ons bespreek dan die prospektus vir vordering op die kort termyn in spesifieke areas van ondersoek binne hierdie veld, insluitend die potensiaal van nuwe KI-metodes wat nog nie toegepas is op die studie van morfologiese evolusie nie. Ons let veral op sleutelareas waar KI onderbenut bly en gebruik kan word om studies van evolusionêre morfologie te verbeter. Hierdie kombinasie van huidige metodes en potensiële ontwikkelings het die vermoë om die evolusionêre analise van die organismefenotipe in evolusionêre fenomika te omskep, wat lei tot 'n era van “big data” wat die studie van fenotipes in lyn bring met genomika en ander areas van bioinformatika.

## Introduction

The rapid proliferation of tools using artificial intelligence (AI) has highlighted both its immense potential and the numerous challenges its implementation faces in biological sciences. Traditional AI methods (i.e., machine learning) have been widely used in biology for decades; indeed, common analytical methods such as principal component analysis (PCA) and cluster analysis are both types of machine learning (ML). Since the early 2010s, deep learning (DL) has gained significant traction and is increasingly applied to biological problems, including image analysis (Angermueller et al. 2016; Moen et al. 2019; Hallou et al. 2021; Liu et al. 2021b; Pratapa et al. 2021; Akçakaya et al. 2022; Ravindran 2022; Li et al. 2023) and molecular analysis (Atz et al. 2021; Kuhn et al. 2021; Kwon et al. 2021; Audagnotto et al. 2022; Korfmann et al. 2023), among other broad topics within ecology and evolutionary biology (Lürig et al. 2021; Borowiec et al. 2022; Pichler and Hartig 2023).

One key area of biological inquiry relevant to diverse topics is the field of evolutionary morphology, which aims to characterize and reconstruct the evolution of organismal phenotypes (e.g., Alberch et al. 1979; Love 2003). The scope of evolutionary morphology is vast, encompassing pattern, process, and mechanism, from cellular to macroevolutionary levels, across the entire 3.7-billion-year history of life on Earth and, consequently, often involves large datasets (e.g., Cooney et al. 2017, 2019; Price et al. 2019; Goswami and Clavel 2024; Hoyal Cuthill et al. 2024). Comparative evolutionary analyses in particular require large sample sizes for robustness in statistical analysis or evolutionary modeling (e.g., Cardini and Elton 2007; Guillerme and Cooper 2016a, 2016b). Researchers commonly face a trade-off between the breadth and depth of their study, as, typically, high-resolution morphological datasets must sacrifice taxonomic, ecological, or chronological coverage owing to time or computational limitations (e.g., Bardua et al. 2019a; Goswami et al. 2019; Rummel et al. 2024). AI offers an unparalleled opportunity to bridge this breadth–depth gap and thus transform the field into “big data” science, thereby supporting the development of evolutionary morphology. By making large-scale data analysis more feasible, integrating AI into this field will ultimately allow a better understanding of the drivers and mechanisms of morphological evolution.

Here, we focus on the applications of AI to the study of evolutionary morphology, exploring not only preexisting uses but also the potential of recently developed AI methods that have not yet been applied to the study of morphological evolution. We introduce the main available AI techniques, categorizing them into three groups based on their order of appearance: (1) ML, (2) DL, and (3) recent advancements in DL from transformers to large-scale models. Next, we present existing AI approaches in the order of a common lifecycle of evolutionary morphological studies: (1) data acquisition, (2) image data processing, (3) phenomics, and (1) evolutionary analysis. We also focus on 10 case studies in which AI can benefit evolutionary morphological studies, and provide a table of AI tools already available that can be integrated into evolutionary morphology research. Finally, we discuss the areas where there are potential, but limited current, applications of AI to key areas in evolutionary morphology.

## Evolution of AI methods

**Fig. 1:**
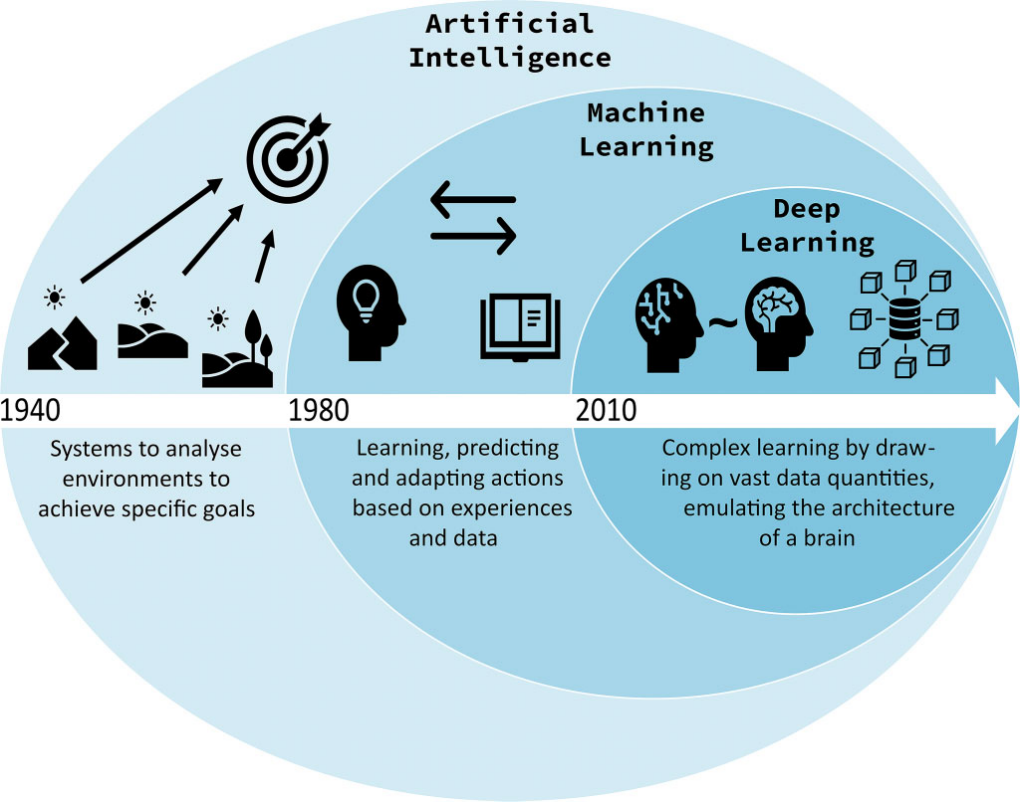
Broad definitions, relationships, and differences between artificial intelligence, machine learning, and deep learning, the sequential development of each successive subset, and their broad introductions over time (Carbonell et al. 1983; Goodfellow et al. 2016).

We begin by providing the key definitions necessary for a base-level understanding of this review. These primarily center on the nested relationships of AI, ML, and DL (Fig. 1), but also include the adjacent and overlapping field of computer vision. Because AI applications for evolutionary morphology primarily involve the analysis of images or text, computer vision is often an integral part of AI applications to evolutionary morphology, including most of those discussed here. However, it is worth noting that computer vision is not limited to AI but also present in numerous applications for image data that do not involve AI (e.g., Samoili et al. 2020). Further methodological definitions are provided where required in the main text.

***Artificial intelligence***, or AI, is particularly challenging to define, as its scope is extremely broad. The European AI strategy (European Commission 2018) provides a definition as follows: “Artificial Intelligence refers to systems that display intelligent behaviour by analysing their environment and taking action—with some degree of autonomy—to achieve specific goals,” leaving the interpretation of intelligent behavior open to the reader. Russell and Norvig (2021) provide a more operative definition of AI, as a system that can either “reason,” act human-like, or act rationally.
***Machine learning***, or ML, is a subset of AI that can be defined as “the ability of systems to automatically learn, decide, predict, adapt, and react to changes, improving from experience and data, without being explicitly programmed” (Amalfitano et al. 2024).
***Neural networks*** are ML models consisting of layers of interconnected nodes (neurons). Each neuron receives the input, processes it using mathematical functions with parameters, and passes the output to the next layer, enabling the network to learn patterns and make decisions based on data.
***Deep learning***, or DL, is a branch of ML wherein learning is achieved through complex neural networks with many layers and parameters. With a large number of parameters, DL models are able to make predictions on difficult tasks or extract features with data that have complex structures.
***Computer vision*** is a multidisciplinary field of computer science that enables machines to interpret, analyze, and understand visual information from the world, through image and video processing algorithms. It refers to using computers for pattern recognition in two-dimensional (2D) and three-dimensional (3D) digital media. While many applications of computer vision for evolutionary morphology involve AI, it is not limited to AI and is applied in diverse fields.

### Classical machine learning

Prior to the development of DL, ML methods had been successfully used for classifying, clustering, and predicting structured data, such as tabular data. Techniques like random forests (Breiman 2001) and K-means clustering (MacQueen 1967) have been widely used in evolutionary morphology studies (Dhanachandra et al. 2015; Pinheiro et al. 2022). These methods are typically end-to-end, whereby data are inputted, and the methods learn patterns to generate results. Meanwhile, when it comes to image data, classical computer vision pipelines were composed of two separate computational steps. The first involved the extraction of local or global characteristics (features) that were deemed useful for a task from images. This meant that, for example, the borders and edges of an image needed to be identified, and subsequently, an object could be detected based on the edges, as in the active contours (Kass et al. 1988) and level set methods (Osher and Sethian 1988; Chan and Vese 1999). The extracted features were then used as inputs to ML algorithms that were optimized for structured data.

Subsequent efforts were devoted to the design of methods to extract relevant structures within an image, such as Haar features (Papageorgiou et al. 1998), scale-invariant feature transform (Lowe 2004), histogram of oriented gradients (Dalal and Triggs 2005), Fisher kernels (Perronnin and Dance 2007; Perronnin et al. 2010), and curvelets (Candès et al. 2006). These engineered (or hand-crafted, or heuristic) features were then often used as inputs for ML methods, which can be broadly classified into the following approaches: classification, clustering, and dimension reduction (Lloyd 1982; Cortes and Vapnik 1995; Breiman 2001; Jolliffe and Cadima 2016). Although DL architectures and convolutional neural networks (CNNs) had already been proposed in the early 1990s (LeCun et al. 1989), their success was limited due to a lack of computational power and the availability of large datasets needed to fully exploit their capabilities. However, there were some attempts to design ML systems that could learn the extraction of optimal linear features for downstream tasks (e.g., classification, detection, and clustering) within a boosting framework (Vedaldi et al. 2007).

### Deep learning

Although artificial neurons (McCulloch and Pitts 1943) and then artificial neural networks (Rosenblatt 1958) were introduced several decades ago, they were often outperformed by other methods, especially ensembles of decision trees like random forests (Breiman 2001) or boosted trees (Chen and Guestrin 2016) across a variety of tasks at that time. This was mainly due to the difficulty in training fully connected networks (wherein the neurons of each layer are connected to all neurons in the following layer) with more than a few layers. Even when shared-weights approaches and CNNs were introduced (Fukushima 1980; LeCun et al. 1989), they remained on the fringe of the AI community, with the primary bottlenecks being the computational power required to build networks with multiple layers and the amount of data needed to train such systems.

As the availability of data and the performance of computer hardware improved, especially with the advent of graphics processing units (GPUs), deep CNNs rose to prominence in the field of computer vision. A key turning point was reached in 2012, when a deep CNN achieved the best result in the ImageNet Large Scale Visual Recognition Challenge (classifying millions of images into thousands of classes) (Krizhevsky et al. 2017). Ever since, computer vision tasks have been dominated by solutions using deep neural networks (DNNs; a key DL technique), to the extent that learning with DNNs is now generally referred to as AI, a name formerly used only for methods trying to solve general intelligence tasks, rather than specific tasks. In recent years, DL has undergone significant expansion into diverse domains, demonstrating its adaptability and offering promising solutions to challenges in various fields such as physics, medicine, and even gaming (Silver et al. 2016; Shallue and Vanderburg 2018; Raissi et al. 2019; Poon et al. 2023). Concurrently, neural network-based methods such as long short-term memory (LSTM) (Hochreiter and Schmidhuber 1996) and recurrent neural networks (RNNs) (Graves et al. 2013) have been applied to sequential data and have shown great results for handling text and time series data, which has led to them being widely used in natural language processing (NLP) tasks (Zhou et al. 2015; Canizo et al. 2019).

The difficulty of gathering a large enough dataset to fully train a DL model for a specific task can be mitigated by the assumption that many low-level features learned by large models are generally enough for most tasks (Tan et al. 2018). Under this assumption, the features learned for a task can also be transferred for a different task. A technique frequently used in DL is the use of pretrained models that are then fine-tuned (the entire model adapts to the new task) or used for transfer learning (only the final layers of the models are trained) (e.g., Mathis et al. 2018). Using pretrained models reduces the need for large datasets, often improves model performance, and saves training time and resources (Devlin et al. 2019; Dosovitskiy et al. 2021). A common example is the use of models pretrained with the ImageNet dataset​ for downstream tasks (Ren et al. 2016; Chen et al. 2017), such as in Sun et al. (2018), where the ImageNet-based model was used for object detection from underwater videos in marine ecology.

### Transformer, large-scale AI models, and multimodal learning

In 2017, a model architecture known as Transformer was developed to address many NLP tasks, such as translation (Vaswani et al. 2017; Vydana et al. 2021). Transformer uses a self-attention mechanism, allowing each token (i.e., words, phrases, sentences, etc.) to interact with other tokens during training. Transformer can handle more information than RNNs and LSTM, can analyze contextual information, and is also better at parallelization. Since Transformer's introduction, it has become state-of-the-art for many NLP tasks (Ahmed et al. 2017; Baevski and Auli 2019). By 2020, most computer vision models were using CNN-based methods. Transformers have been implemented as the backbone architecture for computer vision models (Dosovitskiy et al. 2021; Liu et al. 2021c). A common method is to divide an image into patches, which are treated as sequential inputs similar to tokens in NLP tasks (Dosovitskiy et al. 2021). When Transformer is applied, models can recognize patterns and relationships between different image parts.

**Fig. 2:**
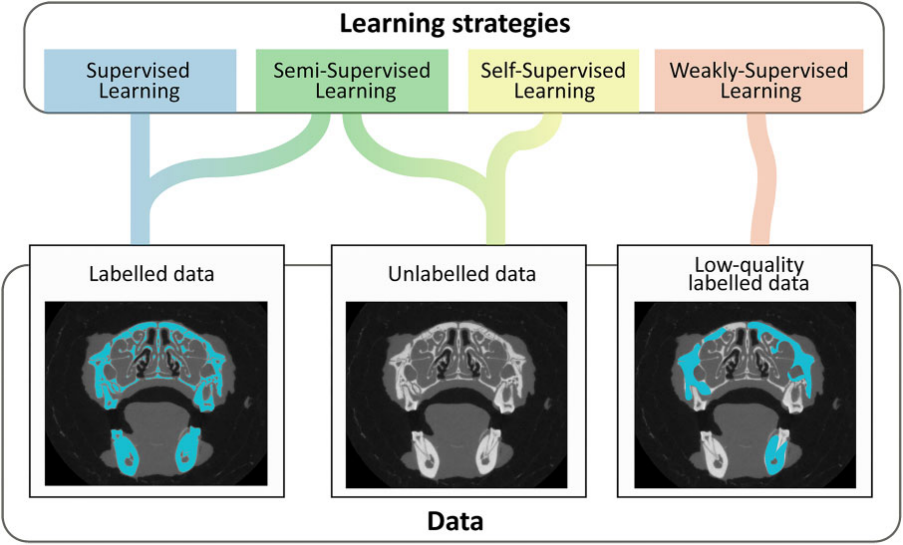
An overview of existing learning strategies and the levels of labeling used in these strategies.

Research has shown that having large and diverse datasets allows models to generalize well and perform more accurately (Russakovsky et al. 2015; Goodfellow et al. 2016). Supervised learning is a common learning strategy that requires all training data to be manually labeled. However, gathering a sufficient quantity of labeled data is often extremely labor-intensive, as most applications require relatively large training datasets. Different training strategies are applied to tackle this problem (Fig. 2). Semi-supervised learning uses both labeled and unlabeled data for training (Zhu and Goldberg 2022), weakly supervised learning uses less accurately labeled data for training (Lin et al. 2016), and self-supervised learning only uses unlabeled data (He et al. 2021). These strategies allow DL models to leverage as much data as possible without the need for extensive manual work.

Self-supervised learning has been widely used in NLP studies. One example uses parts of sentences as input data to predict entire sentences, thereby allowing all the unlabeled text to be considered as training data (Devlin et al. 2019). Models trained with masked sentences can be used as powerful pretrained models for fine-tuning downstream tasks. With access to more training data and larger model architectures, generative models like the Generative Pre-trained Transformer (GPT) family were developed (Radford et al. 2018, 2019; Brown et al. 2020). Recent GPT models (e.g., GPT-3.5 and GPT-4) are capable of performing exceptionally well on many NLP tasks, even when doing zero-shot (no training needed for new tasks) or few-shot (only a few training samples needed) learning (e.g., Brown et al. 2020).

Contrastive learning is one of the self-supervised learning strategies that is widely used in computer vision (Wu et al. 2018; Oord et al. 2019). The idea of contrastive learning is to train a model to map similar instances (e.g., a different view of the same image) close together while mapping dissimilar images farther apart in the feature space. Although different approaches have been designed to map similar/dissimilar instances (Chen et al. 2020; He et al. 2020), the fundamental concept remains the same. As a result, contrastive learning enables models to capture intricate visual patterns and semantics from data without the need for labeled data, thereby improving performance on downstream tasks. Later, masked images (where parts of images are obscured) have been used to predict original images and have been shown to achieve promising results (He et al. 2021).

These learning strategies enable the training of large models using unlabeled or a small set of labeled data, which is particularly applicable to biological sciences, given the wealth of data available in natural history collections (Johnson et al. 2023). Additionally, AI has been successfully applied to process various data modalities, including text, images, and videos (Radford et al. 2021). Multimodal learning can be implemented by combining features extracted from different data modalities into one feature space. This enables tasks such as generating images with text descriptions or generating descriptions for images (Radford et al. 2021). With more data available (e.g., through self-supervised learning) and the advancement of AI models (e.g., Transformer), the field of multimodal learning is rapidly evolving. There have been a few implementations of multimodal models on biological data (Stevens et al. 2024). In evolutionary morphology, multimodal learning can effectively process diverse data modalities, such as photographs, micro-computed tomography (micro-CT) scans, and 3D mesh models.

A full review of the aforementioned three major stages in the development of AI is beyond the scope of this paper, and there are numerous other subfields of AI not explicitly reviewed in this section, such as robotics (Dumiak 2008) and graph neural networks (Dettmers et al. 2018). Nonetheless, these methods hold substantial potential for the study of evolutionary morphology and, where appropriate, will be noted in the subsequent sections focused on current usage and future applications in this field.

## AI for evolutionary morphology

**Fig. 3:**
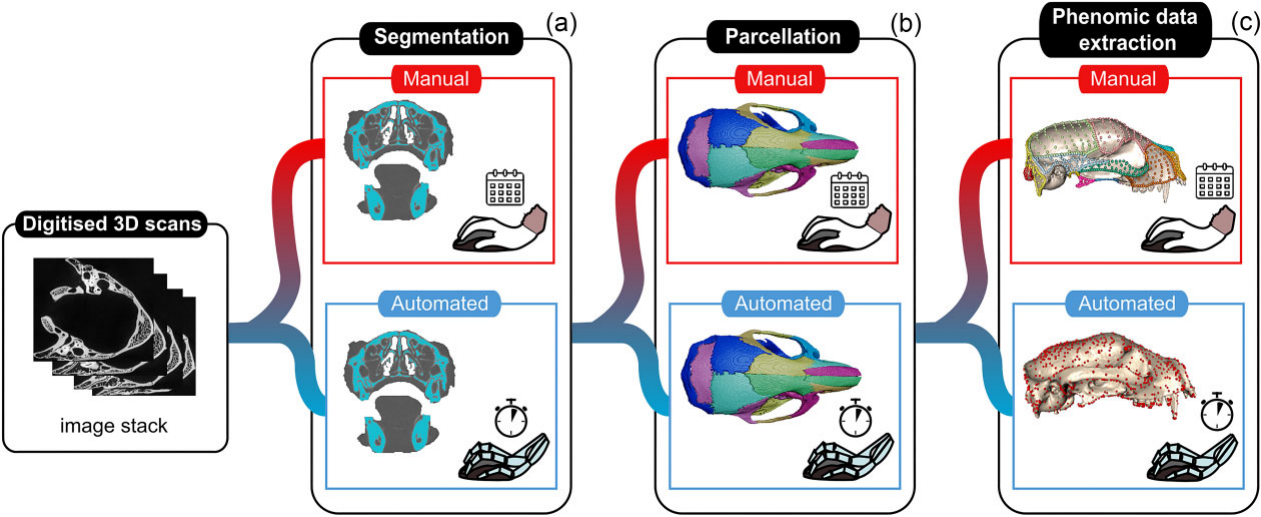
Schematic of a common workflow using manual and AI approaches for evolutionary morphological analysis involving 3D images and meshes. The main steps are: (**a**) segmenting the specimen from the background; (**b**) isolating the scan into target regions; and (**c**) extracting phenomic data from the isolated regions.

In this next section, we pivot toward a goal-oriented review and prospectus of applications of AI in evolutionary morphology, with accompanying case studies. We present the overview of currently available AI tools for evolutionary morphology studies in four sections: data acquisition, image data processing, phenomics, and evolutionary analysis. We introduce these methods with a schematic of generalized AI workflows (Fig. 3), which are expanded in the following sections.

### Data acquisition

The first step of acquiring data is to collect the relevant samples, which are to be used in the subsequent investigation under appropriate best practices (e.g., Parham et al. 2012). For analysis of evolutionary morphology, this includes obtaining not only the data that are being measured but also the corresponding metadata such as details about museum specimens (e.g., Smith and Blagoderov 2012, Davies et al. 2017, Ioannides et al. 2017, Johnson et al. 2023). The suitability, quality, and quantity of data are of critical importance to the development and implementation of AI models. Data should be diverse and clean; fulfilling these requirements can make a larger difference than model choice, and without data that conforms to these requirements, good models will perform badly (Whang et al. 2023). Diverse data include enough examples of each class of interest. Determining how much data is enough depends on the specific problem at hand. Scarce data can be expanded using existing databases or by employing pretrained networks for transfer learning (Sharif Razavian et al. 2014). However, DL models can be successful on small training sets. Few-shot learning is a form of transfer learning that uses training data where 1–20 examples of each class are available (Wang et al. 2021b). Scarce data tend to have an imbalance between the presence of classes in the dataset; the model may find it difficult to discriminate the scarcely represented classes and perform unreliably (Schneider et al. 2020). Clean data minimize errors from training datasets. Preprocessing a dataset increases the suitability of the data for training and can include contrast enhancement, noise reduction, and masking, where a portion of the image is designated for further analysis (Lürig et al. 2021).

Data scarcity and imbalance can also be improved by additional data collection or artificial data expansion (e.g., data augmentation). Alternatively, an imbalance can be tackled by explicitly accounting for biases in the training algorithm (Buda et al. 2018). Augmentation effectively increases the size of the training set without new data collection by manipulating images to create “new” images from the existing data. This can be achieved by rotating, mirroring, scaling, or altering the pixel values (Shorten and Khoshgoftaar 2019; Mulqueeney et al. 2024a). This process must be controlled with the aim of the model in mind. For example, for planktonic foraminifera, the chirality of a species can be important in species classification, meaning augmentation by mirroring (i.e., horizontally flip) makes the labeled image into a facsimile of a different species (Hsiang et al. 2019).

#### Identifying and cataloguing specimen data

Many, perhaps even most, studies of evolutionary morphology are based primarily on data housed within museum collections. However, museum collections are rarely fully catalogued and searching for a specific specimen or representatives of specific groups can be challenging. This difficulty is because data are often inconsistent in quality and structure, particularly in large collections (Dutia and Stack 2021). Some of the key challenges to address in cataloguing museum specimens include recognizing species and extracting taxonomic and metadata to enable effective searches. AI can play a key role in this, particularly when it comes to tasks of digitizing, identifying, cataloguing, and locating specimens within collections.

At its most basic definition, digitization involves the creation of digital objects from physical items. Within museums, this is often attributed to the photographing, scanning, or filming of physical specimens (Blagoderov et al. 2012). However, traditional ways of digitizing artifacts, such as digitizing each specimen individually, can be invasive to the specimen, time-consuming, and not very cost-effective (Price et al. 2018). This has led to a series of innovations that can help advance museum digitization, from drawer scanning (Schmidt et al. 2012), which enables multiple specimens to be digitized at once, to special rotating platforms that, when combined with photogrammetry techniques, allow for the 3D scanning of specimens, while avoiding the use of more expensive or time-consuming scanning techniques, as seen in Ströbel et al. (2018) and Medina et al. (2020). ML can lend a hand to these innovations to advance digitization even more, such as the use of computer vision techniques and CNNs to segment individual specimens from whole-drawer scans (Blagoderov et al. 2012; Hudson et al. 2015; Hansen et al. 2020).

DL has recently been applied to many types of biological specimens and collections (e.g., Soltis et al. 2020). These methods have been developed and applied extensively to recognize species, metadata, traits, and even life history stages of digitized specimens. This is most established in the botanical sciences, where flat herbarium sheets are easily digitized in large numbers as 2D photographs (Gehan et al. 2017; Goëau et al. 2022). This has also led to advances in DL-based classification and segmentation tasks of traits within digitized herbarium sheets (e.g., Weaver et al. 2020; Walker et al. 2022), such as with LeafMachine (Weaver and Smith 2023), which is a tool for automatic plant trait extraction, from flowers and leaves to rulers and plant label detection. In some instances, albeit to a lesser degree, species identification methods have also been applied to digitized photographs of animal collections (e.g., Macleod 2017; Ling et al. 2023). Applications of DL to species identification of both plants and animals from photographs have been greatly enhanced by citizen science, resulting in useful online tools such as iNaturalist (Unger et al. 2021) and Pl@ntNet (Goëau et al. 2013). CNN algorithms have borne promising results and can correctly distinguish morphologically similar species (Feng et al. 2021; Hollister et al. 2023). Other ML methods, such as those described by Wilson et al. (2023), have also been applied to rescaling and increasing the quality of and extracting metadata from images of museum specimens, allowing for automatic feeding of this information into databases.

Beyond images of the specimens themselves, AI approaches for capturing information from specimen labels can save vast amounts of manual effort for cataloguing specimens and making key data searchable (Case Study 1). Together, species identification and taxonomic and metadata extraction methods from images represent a powerful tool for unlocking the full potential of natural history collections. These approaches can make data more discoverable and usable for documenting biodiversity both in collections and in the field (Schuettpelz et al. 2017; Wäldchen and Mäder 2018; White et al. 2020; Karnani et al. 2022).

Information on specimens is not limited to museum catalogues but is also available in the wealth of scientific publications detailing and imaging specimens for varied purposes. However, extracting taxonomic data from the literature to describe or identify living and fossil species is a time-consuming task. It may also be difficult to find the first appearance of a species name and correctly identify all synonyms for a taxon, as well as accounting for more recent taxonomic reclassifications. Recently, a few research groups have attempted to tackle this problem using ML, with both NLP and other DNN algorithms successfully applied to extract scientific terms and taxonomic names from scientific articles (e.g., Le Guillarme and Thuiller 2022). This is a relatively new application of ML, and more work is required to train models on a variety of sources, including articles in different languages and historic publications.

Once these data are captured, we need effective tools to search for connected specimens. ML has not yet been adopted on a large enough scale to allow searching global natural history collections and connecting specimens. For instance, Dutia and Stack (2021) created “Heritage Connector,” a toolkit for using ML to allow better connectivity of specimens in collections and publications. This software achieved a precision score of greater than 85% with science museum records of 282,259 objects from 7743 organizations. Further development of software such as this, applied on a global scale, will improve access to the vast specimen data held in natural history collections worldwide.

## Case Study 1: Machine learning within museum digitization and data collection

(A) Label extraction within digitization pipelines

**Fig. 4:**
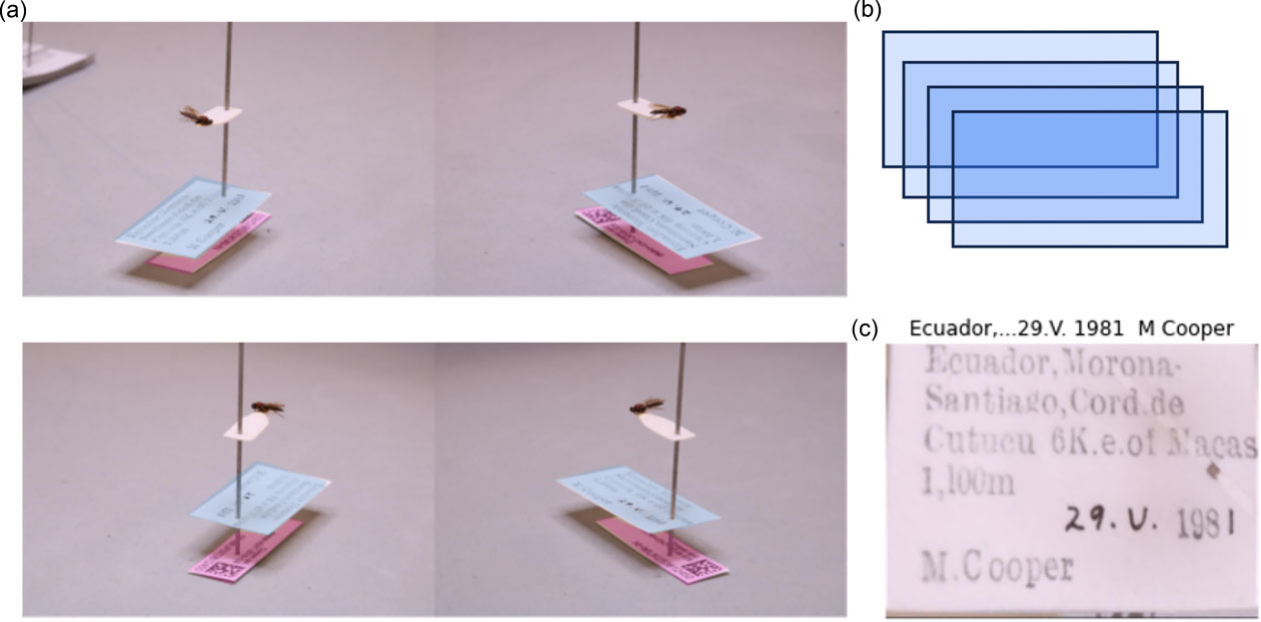
An example of the workflow described in Salili-James et al. (2022b): (**a**) using a setup introduced in Price et al. (2018), the algorithm uses a CNN model to segment all labels found on each of the four images of the specimen, (**b**) for each label, it then merges the four layers together in order to have one version of each label, which can be fed into an automatic transcription algorithm using OCR, and (**c**) an example of a merged label, with a sample of the automatically transcribed text above it.

ML tools, along with the latest digitization innovations, have allowed for the development of techniques that enable digitizers to extract information from labels automatically while digitizing specimens. For example, a cost-effective and efficient pinned insect digitization process was introduced by Price et al. (2018), which involved placing the specimen within a light box and capturing a handful of photographs simultaneously with multiple cameras from varying angles. The framework described there and in Salili-James et al. (2022b) shows how one can turn to ML to merge labels together from the differently angled images to obtain clean, unobstructed images of labels and hence automatically extract textual information from them for digitization purposes (Fig. 4). The first step in this process is reliant on DL tools such as CNNs to locate labels from the multiple images of the specimen. Next, various mathematical and computer vision tools are used to “stitch” the found labels together to have one clear image of each label. These labels can then be fed into an optical character recognition (OCR) and then an NLP algorithm to transcribe the text and to automatically obtain trait information. This leads to a streamlined, automated pipeline to extract label information that helps speed up digitization efforts.

(B) Knowledge graphs within digitization pipelines

**Fig. 5:**
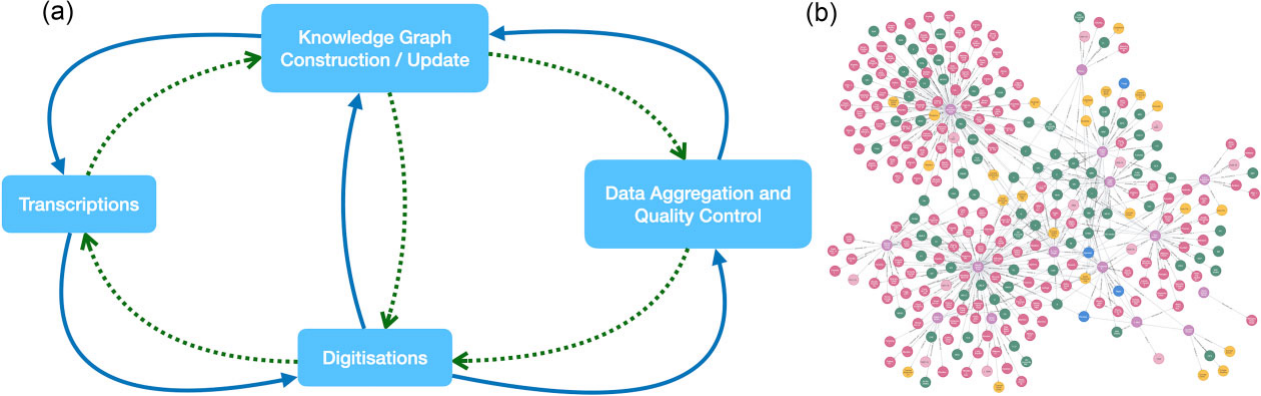
An example of (**a**) the facets of natural history collection data making up a node of a knowledge graph and (**b**) a whole knowledge graph showing interconnectivity between nodes.

Transcribing text from labels is one step of the data extraction process, but understanding the textual data and conceptualizing the data and specimen within a larger picture is another, much bigger goal, which may one day be accomplished with knowledge graphs (Dettmers et al. 2018). Knowledge graphs are structured and contextual data models that represent semantic information about concepts, entities, relationships, and events (Fig. 5). Broadly, this can lead to representations of data structured in graphs with interlinking entities, allowing users to define relationships between different items within large datasets. The Natural History Museum, London, has recently begun developing knowledge graphs, with an initial focus on herbarium sheets (Gu et al. 2022). Herbarium sheets contain a wealth of information from handwritten and printed labels appended to the sheet, to handwritten text containing trait and specimen information written directly on the herbarium sheet. Knowledge graphs can help determine links between herbarium sheet specimens, and subsequently, combined with other ML techniques, can be embedded into digitization workflows (Gu et al. 2023). For example, with integrated OCR tools, incorrect or missing information can be corrected and filled in, by analyzing the relationships present in the graphs. Knowledge graphs can be used to identify data outliers, find historical errors, and clean data, making them a great tool for digitization.

### Image and scan data collection

While we refer to the use of images for specimen cataloguing earlier, here we focus on the details of image data collection for analysis. The use of images is central to the study of evolutionary morphology, from simple drawings and photographs to computed tomography (CT) scans (Cunningham et al. 2014). Two-dimensional digitization often involves photographing collections (i.e., specimens, drawers, etc.), whereas three-dimensional digitization involves generating images of specimens using techniques such as photogrammetry, surface scanning, or volumetric scanning.

Present-day efforts to digitize specimens with 2D images for large-scale data acquisition and utilization often involve some automated processes, which can streamline both digitization and the interpretation of data (Case Study 2). Recent studies (Scott and Livermore 2021; Salili-James et al. 2022b) describe software that uses ML models to identify regions of interest (ROIs) in 2D images. Once trained, AI software can capture photographs, segment ROIs, and complete other tasks for large collection datasets. This streamlines the overall acquisition and processing of digital data. Over time, ML software becomes more accurate and efficient as it learns through training datasets and is exposed to more data.

The ability to generate high-resolution 3D images has increased exponentially in recent years, particularly with initiatives for mass scanning of collections and databases for open sharing of image data, including DigiMorph (Rowe 2002), Phenome10K (Goswami 2015), and MorphoSource (Boyer et al. 2016). These images can then undergo segmentation or region identification and data extraction, where specific components are identified and separated from the image for further processing or evaluation.

Novel and potentially more efficient scanning methods are continuously emerging. For instance, a neural radiance field (NeRF) is a fully connected neural network that can generate a 3D scan of an object by inputting photographs of it from different viewpoints (Martin-Brualla et al. 2021). Compared with traditional photogrammetry and CT scanning, this method is able to compute 3D scans based only on sparse images (Yu et al. 2021). While the resolution and accuracy are typically inferior to a full 3D scan, it can make 3D data capture more accessible and faster for some objects (e.g., extremely large specimens).

## Case Study 2: Robotics for digitization

(A) Machine learning and robotics for specimen digitization

**Fig. 6:**
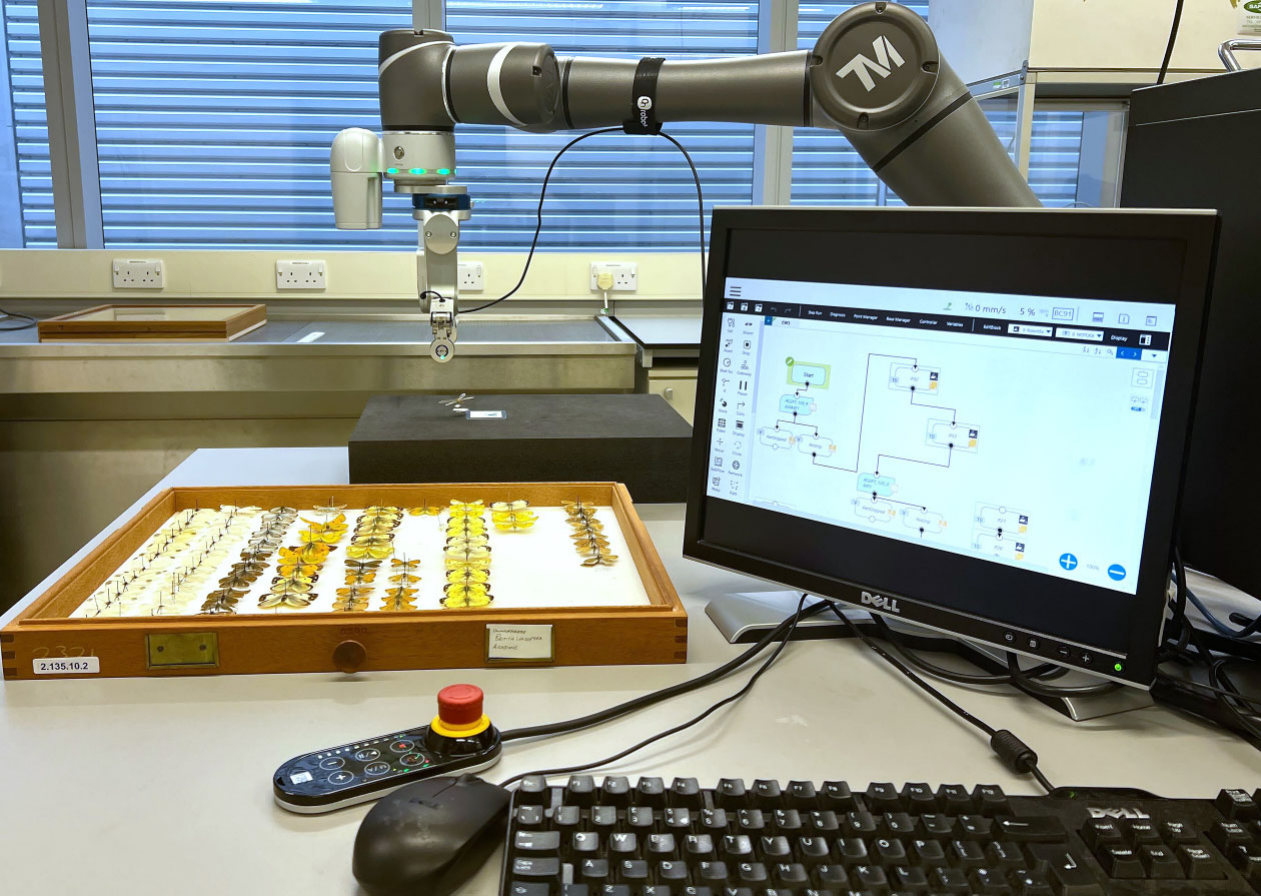
A Techman 500 robotic arm in action at the Natural History Museum, London, placing down a sample pinned specimen from a Lepidoptera collection. Here, the robotic arm has been trained to locate the specimen from the drawer, and then pick it up and place it on a board in order to scan the specimen. Synthetic specimens were used in the training stage for this task.

One technological advancement that can aid digitization is robotics; robots are indeed already in use in other sectors, such as book scanning at libraries (Dumiak 2008). Though usually highly expensive, the prices of robotic arms have been decreasing (Zhang et al. 2022), and one can now purchase a robotic arm for less than £20,000 (Stanford University 2022). This has enabled digitization teams within museums such as the Natural History Museum, London, to start exploring robotics for digitization research (Scott et al. 2023). Here, the goal was to have a collaborative robot (cobot) aid a digitizer in the mass digitization of certain specimens (Fig. 6). Computer vision can be combined with robotics in order to identify, move, and scan specimens, with synthetic specimens used during the training stages in order to mitigate the risks of specimen damage. Thereafter, by implementing CNN algorithms and/or turning to reinforcement learning, a robotic arm can lead to a pipeline that can enable digitization teams to mass digitize multitudes of specimens, even possibly overnight, revolutionizing museum digitization work.

(B) Automation of specimen digitization

The use of automated robotics for digitization and high-throughput data collection has historically been applied to 2D methods such as photography. Three-dimensional data, such as micro-CT data, can also be collected with new robotic technologies like autoloaders (Rau et al. 2021). Autoloaders allow users to set up multiple specimens for micro-CT and synchrotron scanning, set distinct parameters for each scan, and subsequently run the autoloader without supervision. The autoloader processes specimens in a queue, pulling each from the stand using a robotic arm, and setting up distinct parameters for each (van de Kamp et al. 2018; Rau et al. 2021). This fully automated process results in greater acquisition efficiency, as the number of specimens digitized via this method increases when digitization can occur without technician supervision. While the use of robotic technology to digitize collections can greatly increase the efficiency of image collection, the improvements are more than mechanical. Robots can learn behaviors through reinforcement learning (trial and error, as well as rewarding and/or punishing). By interacting with the environment (e.g., the digitization room), robots can learn optimal actions that maximize rewards (e.g., successfully imaging a specimen).

### Image data processing

Capturing image data has become increasingly widespread in recent years, with large programs focused on mass scanning of natural history collections (Hedrick et al. 2020). The bottleneck has now shifted to processing images in order to obtain usable phenotypic data. Here, we focus on the major aspects of image data processing: segmentation for feature extraction and element isolation for both 2D and 3D data.

Image segmentation refers to dividing an image into meaningful areas or objects and extracting ROIs, allowing for targeted analysis, and understanding of visual content (Yu et al. 2024). There are two main parts to segmentation: semantic segmentation, where all objects of a class are grouped as one entity when segmented; and instance segmentation, where objects of the same class are distinguished. These types of segmentation facilitate numerous computer vision tasks, including object recognition by isolating objects or regions within an image (Garcia-Garcia et al. 2018; Jin et al. 2022), object tracking (Zhao et al. 2021), and interpreting a scene with multiple objects (Byeon et al. 2015). This process has traditionally been performed without DL (Otsu 1979; Najman and Schmitt 1994; Boykov et al. 1999; Nock and Nielsen 2004; Dhanachandra et al. 2015; Minaee and Wang 2019); however, it remains subjective (Joskowicz et al. 2019) and time-intensive (Hughes et al. 2022). However, in recent years, novel DL methods have facilitated models to achieve high accuracy rates on common benchmarks (LeCun et al. 2015; Kale and Thorat 2021; Luo et al. 2021; Zhao et al. 2021; Yu et al. 2022). DL-based segmentation methods are state-of-the-art for many image segmentation challenges and often outperform other methods.

#### 2D image segmentation

There are many applications of automated methods on 2D image datasets for morphological studies. Many studies have aimed to extract features such as size (Al-Kofahi et al. 2018), shape (Schwartz and Alfaro 2021; Lürig 2022), and pixel values (Van Den Berg et al. 2020; He et al. 2022) from segmented photographic images, and used the extracted features to quantify organismal morphology. Moreover, due to the properties of 2D radiological images (e.g., magnetic resonance imaging [MRI] and CT), such as distinguishable grayscale values, specific segmentation models have been developed, particularly for applications to medical images (Ronneberger et al. 2015). Some studies have used these models to segment these radiological images and measure morphological features (e.g., Norman et al. 2018; Montagne et al. 2021). As these automated segmentation methods allow for greater consistency among measurements, they make measurements more repeatable, and, particularly in medical fields, they allow for better longitudinal studies (Willers et al. 2021). These methods applied to 2D images can be adopted for studying evolutionary morphology, for instance in evo-devo or histology; however, as an increasing number of investigations seek more detailed measurements, 2D images may lack sufficient spatial or internal structure information, making segmentation on 3D cross-section images crucial.

#### 3D image segmentation

AI approaches to image segmentation have been adapted for 3D data, being routinely applied to image stacks generated from CT (Ait Skourt et al. 2018, Kendrick et al. 2022) and MRI (Milletari et al. 2016; Lösel et al. 2020). In addition, user-friendly tools for segmenting medical images have been developed that offer built-in features for automatic image segmentation such as Dragonfly (Comet Technologies Canada Inc. 2022) and Biomedisa (Lösel et al. 2020). These have since been applied to datasets on biological systems (Lösel et al. 2023; Rolfe et al. 2023; Mulqueeney et al. 2024a) (Case Study 3).

Beyond increasing the efficiency of segmentation over manual thresholding, DL-assisted segmentation may be beneficial whenever thresholding ROIs is not possible. For example, when specimens being scanned are very dense, scans may not have a consistent perceived density (e.g., Alathari 2015; Furat et al. 2019). Another case where DL segmentation may be useful for CT data is when segmenting regions of an object made of the same material (i.e., if an object of a single material ossifies as a single structure but has varying patterns of ossification along the structure) or when multiple objects have similar densities. Objects with similar densities may be displayed at the same grayscale value through the scan, thus may be difficult to distinguish. Scans such as these are often also very noisy as a result of the high power of the beam needed to penetrate them, frequently resulting in artifacts and irregularities within images (Das et al. 2022). DL segmentation models can be trained to overcome these issues and segment scans based on visual patterns when a minimal number of slices are prelabeled (Tuladhar et al. 2020). Noteworthy uses of this approach include distinguishing fossils from rock matrices with a comparable composition within CT images (e.g., Yu et al. 2022; Edie et al. 2023), a common problem when imaging paleontological specimens.

## Case Study 3: Image segmentation for volume rendering

**Fig. 7:**
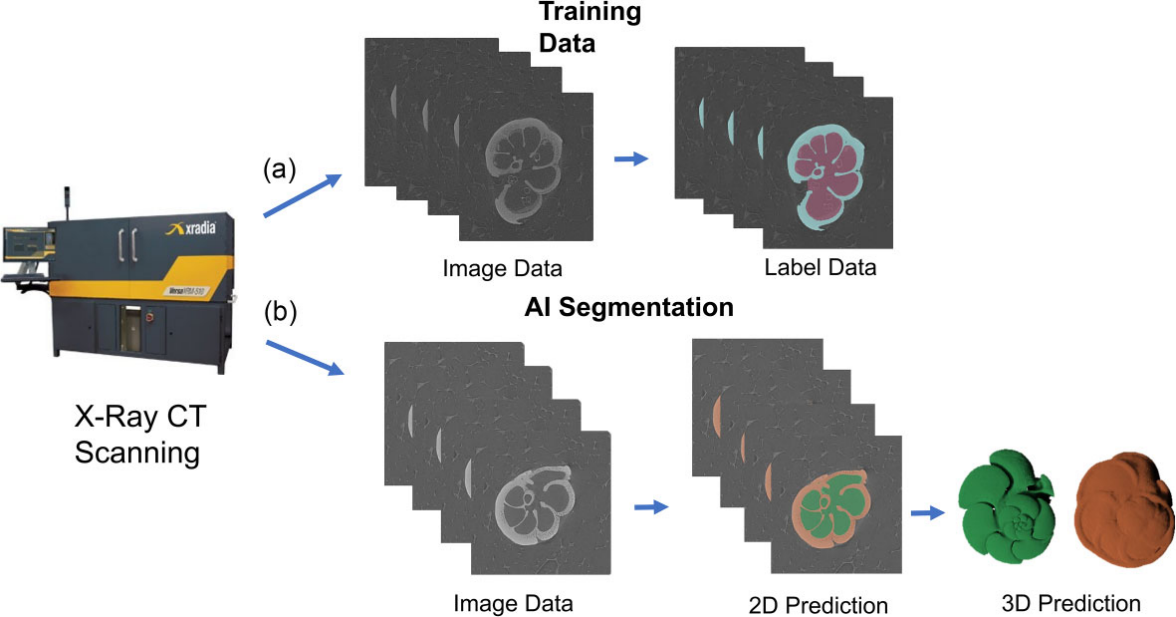
Workflow from Mulqueeney et al. (2024a) for producing training data and applying a CNN to perform automated image segmentation, reproduced under a Creative Commons Attribution 4.0 International License (https://creativecommons.org/licenses/by/4.0/). The workflow includes (**a**) the creation of training data for the input into Biomedisa and (**b**) an example application of the trained CNN to automate the process of generating segmentation (label) data.

DL tools such as Biomedisa (Lösel et al. 2020) have emerged as powerful solutions for automating feature extraction from 3D images (Fig. 7). They offer an efficient alternative to labor-intensive manual image segmentation methods. In the study by Mulqueeney et al. (2024a), a range of different training sets were used to train a CNN in order to segment CT image data of planktic foraminifera, with each of the accuracy of these models then being compared. The results showed that the efficacy of these neural networks was influenced by the quality of input data and the size of the selected training set. In the context of this case study, this is reflected in the ability of different networks to extract specific traits. In the smaller training sets, predicting the volumetric and shape measurements for internal structures presents a greater challenge compared to the external structure, primarily due to sediment infill (Zarkogiannis et al. 2020a, 2020b). However, by increasing the size of the training set through selecting additional specimens or by applying data augmentation, this problem is mitigated. This reaffirms the principle that expanding the training set leads to the production of better DL models (Bardis et al. 2020; Narayana et al. 2020), albeit with diminishing returns as accuracy approaches 100% (Kavzoglu 2009). These findings help to highlight how training sets can be designed for optimal use in precise image segmentation that is applicable for obtaining a wide range of traits.

### Isolating regions

Isolation of regions within a segmented 2D or 3D image allows for more in-depth analysis of specific areas of focus. In 2D analysis, these methods are present in behavioral ecology and neuroscience, where limb tracking of segmented species in video footage is used to infer behavior of individuals (Mathis et al. 2018; Marks et al. 2022). Similar to 2D, 3D semantic segmentation using CNNs has started gaining traction, notably in the field of pathology (Schneider et al. 2021; Rezaeitaleshmahalleh et al. 2023), engineering (Kong and Li 2018; Bhowmick et al. 2020), and materials science (Holm et al. 2020; Zhu et al. 2020), and is similarly useful for evolutionary morphology. For example, extracting individual structures, such as sutures, from micro-CT scans of whole crania allows detailed analysis of their morphology and the factors driving their evolution (Case Study 4).

## Case Study 4: Image segmentation for automatic trait extraction

**Fig. 8:**
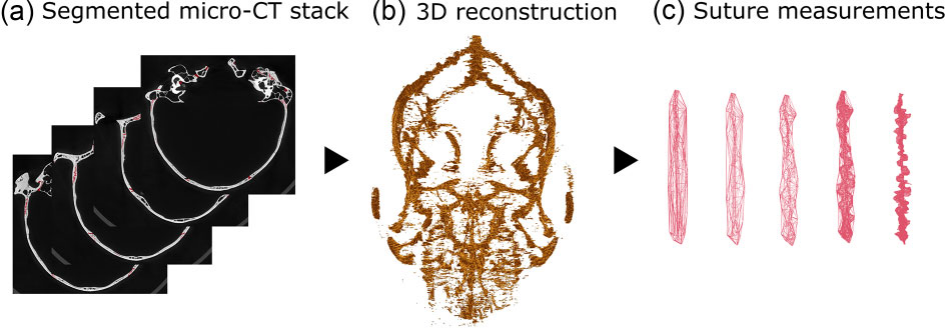
A workflow for extracting sutures on micro-CT scans. This workflow includes (**a**) segmenting sutures on micro-CT scans of mammal skulls. Segmented sutures are used to generate (**b**) 3D reconstructions, which can then be used to calculate (**c**) suture measurements.

Segmentation can also be used to extract phenotypic features directly, such as sutures from their surrounding bones. Cranial sutures are fibrous bands of connective tissue that form the joints between the cranial bones of vertebrates (White et al. 2021). Due to sutures being small regions, measuring them is a highly time-consuming and skill-intensive task. Ongoing work of several authors (M.C., A.G., E.G., Y.H., and O.K.-C.) addresses this methodological challenge using DL models (Fig. 8). First, a dataset was created by segmenting only a specified number of slices (e.g., one out of every 100 slices), which was then split into training and validation sets for model training. Additionally, a test set was created with sutures segmented throughout the entire stack for a few scans, which is used for a robust evaluation of accuracy. As sutures are normally small regions, this creates a class imbalance issue, which can be mitigated through a weighted training approach, with sutures having more weight during training than the surrounding bones. The model performance was evaluated on the test set using the intersection over union for segmented regions. After selecting the best model, sutures for the rest of the scans were predicted and reviewed to generate high-quality suture segmentations. The resulting manually checked and corrected segmentations can be used as a new training set to enhance model performance or used for downstream analysis. Subsequently, features from the extracted sutures can be quantified. Beyond sutures, such a pipeline would be applicable to segmenting (both in 2D and 3D) any open- or close-ended structure, biological or not, that is defined by the interactions between other structures (i.e., cranial endocasts, chambers in mollusc shells, cracks in bones and other materials, and junctions between cells).

In addition to segmentation, isolating regions in 3D meshes is a method of separating scans into biologically meaningful regions (Case Study 5). This approach, however, comes with some important challenges, such as the trade-off between computational cost and the quality of 3D data. Current methods typically employ human-created 3D meshes as benchmarks (Chen et al. 2009), which tend to have low polygon counts and thus do not reflect most biological datasets. As a result, the isolation of regions in 3D meshes has proven challenging, with various methods attempting to overcome quality issues in the CT data (Shu et al. 2022; Sun et al. 2023). For example, work by Schneider et al. (2021) attempted to address this by developing a segmentation pipeline able to process higher-polygon and nonmanifold meshes. This is important for geometric morphometrics, where variations in morphology of focal specimens are only discernible when meshes have sufficient polygons to properly map their topology.

Finally, while feature extraction of known phenotypes from supervised learning is relatively straightforward, it is less clear whether unknown or novel phenotypes are similarly recognizable or whether trained models can accommodate large amounts of variation, both of which will be common in analyses of evolutionary morphology. Nonetheless, applying AI to 3D data with species or features not included in the training set has great potential, particularly in light of promising applications of unsupervised learning to discover unknown phenotypes, for example in cell morphology (Choi et al. 2021).

## Case Study 5: Feature extraction and region isolation on 3D meshes

**Fig. 9:**
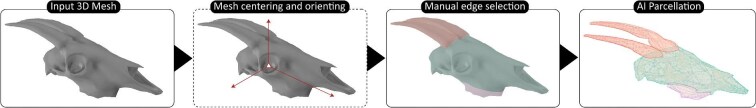
Workflow for segmenting horns/antlers and teeth from a skull using Blender.

In another example from our own work, we sought to conduct a landmark-free 3D morphometric study of skull shape in mammals on 3D meshes, but needed to isolate structures such as cranial ornaments (i.e., antlers and horns) and teeth from the specimen. This is common in analyses using landmarks, as these structures can dominate the variation in an analysis or may have more nonbiological variation due to preservation (i.e., missing teeth). These structures may also warrant their own shape analysis, independent from the skull. To accomplish this, we applied an existing application for accomplishing this task, MedMeshCNN (Schneider et al. 2021), which uses Blender, an open-source 3D software (Blender Online Community 2018). To segregate regions, edges of a mesh are assigned to a specific class (e.g., horns/antlers, teeth, and skull in Fig. 9), resulting in meshes annotated with the ROIs. A model is then trained on the annotated meshes, which can then be applied to other specimens.

### Phenomics

Phenotypes encompass morphology, behavior, development, and physiology, all of which mediate an organism's interactions with other species and its habitat. Phenomics is the organism-wide, high-dimensional extension of the study of phenotype (Houle et al. 2010). Analysis of phenomes thus entails a variety of traits, all of which are essential in understanding the dynamics of organismal evolution, yet the resolution as to which we can currently measure is limited. Here, we discuss how AI techniques can be used to more effectively describe phenotypic traits specific to morphology, with sections related to discrete and meristic traits, univariate measures, shape, color, and pose estimation.

#### Discrete and meristic traits

Morphological traits underpin the study of phenotypic evolution within phylogenetic systematics (Hennig 1966). Discrete and meristic traits are those manually scored by researchers, with discrete data including presence and absence and meristic data referring to counts. Discrete and meristic traits are useful for evolutionary analyses of morphology, evidenced by foundational works of morphological disparity (Foote 1993, 1997; see Goswami and Clavel 2024 for a full review). Discrete traits are also critical for diverse aspects of evolutionary study; for example, they are essential to time-calibrate molecular phylogenies and to reconstruct phylogenetic relations among extinct taxa (Smith and Turner 2005; Lee and Palci 2015). However, morphological traits for phylogenetic applications have many limitations (Lee and Palci 2015), as they can be time-consuming and difficult to collect due to personal interpretations and potential errors (Wiens 2001).

AI tools have shown potential in recognizing and extracting discrete and meristic traits to build morphological matrices for phylogenetic analysis in a quicker and more robust way. AI methods, including CNNs, have been successfully applied on small training datasets to recognize species and extract both discrete and meristic traits (Wäldchen and Mäder 2018). Other examples include using ML tools to extract, classify, and count reproductive structures (Love et al. 2021; Goëau et al. 2022), as well as to produce basic measurements such as leaf size (Weaver et al. 2020; Hussein et al. 2021). These methods have also been shown to work on x-ray scans of fossil leaves (Wilf et al. 2021), including counting stomatal and epidermal cells for paleoclimatic analysis (Zhang et al. 2023). A similar CNN algorithm has also been successfully applied to classify freshwater fish by genera from the Amazon region using photographs of museum specimens, for which traits were recognized with 97% accuracy (Robillard et al. 2023). In animal species traits identification, random forest algorithms have also shown promising results. For example, they performed better than traditional linear discriminant analysis in delimiting between species of snakes from field photographs when given a set of morphological traits (Smart et al. 2021). Overall, these algorithms have the potential to be used in morphological trait extraction and phylogenetic analysis.

#### Univariate measures

Univariate metrics have dominated morphometrics for centuries, but the extraction of univariate traits from a substantial pool of individuals has historically been a laborious and time-consuming process, imposing limitations on available data (Fenberg et al. 2016). Addressing this challenge, AI tools have emerged as effective solutions, streamlining the extraction of univariate traits, including lengths, mass, and size, particularly in 2D images. For instance, neural networks have proven adept at extracting linear measurements, as illustrated by the accurate forewing length extraction of 17,000 specimens of butterflies (Wilson et al. 2023). Moreover, these AI techniques have extended their capabilities beyond simple length measures, such as by measuring plant leaf areas (Kishor Kumar et al. 2017; Mohammadi et al. 2021). Advanced techniques have further enabled the measurement of length across individual anatomical regions, offering a more nuanced understanding than traditional whole-body length measures (Ariede et al. 2023). These techniques have also enabled the extraction of shape proxies, such as ellipticity (Freitas et al. 2023), and the simultaneous analysis of multiple univariate traits (Fernandes et al. 2020).

AI methodologies have seamlessly extended their proficiency from extracting 2D univariate traits to 3D, by employing analogous methods to obtain linear measurements of both length and width within 3D images (Hu et al. 2020; Lu et al. 2023). Some of these methods have the ability to concurrently extract multiple length measurements or features from 3D images (Wu et al. 2021; Yu et al. 2021). Moreover, they can provide volumetric measures of multiple components through segmentation (Lösel et al. 2023; Mulqueeney et al. 2024a).

### Shape

Univariate or linear morphometrics has been a tool in evolutionary morphological analysis for centuries (Zelditch et al. 2004), but recent years have seen an explosion of geometric (landmark-based) and surface morphometrics, greatly increasing the scope for capturing and quantifying organismal shape (Mitteroecker and Schaefer 2022). While surface methods are relatively new, they are expanding rapidly and offer great potential to increase understanding of evolutionary dynamics (Bardua et al. 2019b). Currently, this step is overwhelmingly manual, representing a significant bottleneck for big data phenomic analyses from comparative datasets (Goswami and Clavel 2024). Thus, having automated approaches that can provide high-resolution measures of shape would be hugely influential in allowing large-scale comparative analyses and allowing for more reproducible decisions in trait descriptions.

#### Landmark-based geometric morphometrics

Landmark-based geometric morphometrics is a multivariate methodology, which requires the placement of landmarks that produce 2D or 3D coordinates by labeling homologous anatomical loci to describe biological shapes (Adams et al. 2004; Mitteroecker and Schaefer 2022). Raw coordinates are then transformed using a superimposition method, commonly Procrustes analysis, which uses scaling, rotation, and translation to register objects to a common reference frame so that only biological variation remains (Bookstein 1997). The main advantages of geometric morphometrics include the capacity to densely sample complex shapes in three dimensions: the ability to localize variation, the retention of information on biological homology, and the utility of coordinate data for numerous downstream analyses, from macroevolutionary (e.g., Goswami et al. 2022) to biomechanical analysis (e.g., Pollock et al. 2022). However, the manual placement of landmarks is time-consuming and lacks repeatability (Shearer et al. 2017), especially for big comparative datasets, even when using semi-automated methods (Bardua et al. 2019a).

Automated landmarking techniques have been developed to minimize the user's workload by automating the placement of homologous landmarks. A variety of approaches for automated landmarking via statistical image analysis have been developed in recent decades, frequently relying on image registration to propagate landmarks from one set of scans, or a generic template, to another (Young and Maga 2015; Maga et al. 2017). While these methods have improved in accuracy, they still often lack precision in identifying anatomical loci, even in closely related taxa, particularly around highly variable regions (Devine et al. 2020). Therefore, to improve the obtained results, others have attempted to use DL and computer vision to address the problem of landmark annotation.

One promising approach, currently applied only to 2D images, uses DL for the full process of automatically placing landmarks on specimens (Porto and Voje 2020; Case Study 6). Approaches currently available for 3D images combine image registration and AI, such as Devine et al. (2020), wherein deformable image registration is used to detect the landmarks and then DL is used to optimize their placement, thereby improving accuracy after mapping of landmarks from a template to specimens. Both of these approaches have been shown to reduce both data collection time and error and increase repeatability, thereby supporting phenomic-scale data collection for large datasets. Unfortunately, all present applications behave poorly with even a moderate amount of variation, effectively limiting applications to analysis of conspecifics or congeneric species at present.

### Case Study 6: Geometric morphometrics—automated landmarking

**Fig. 10:**
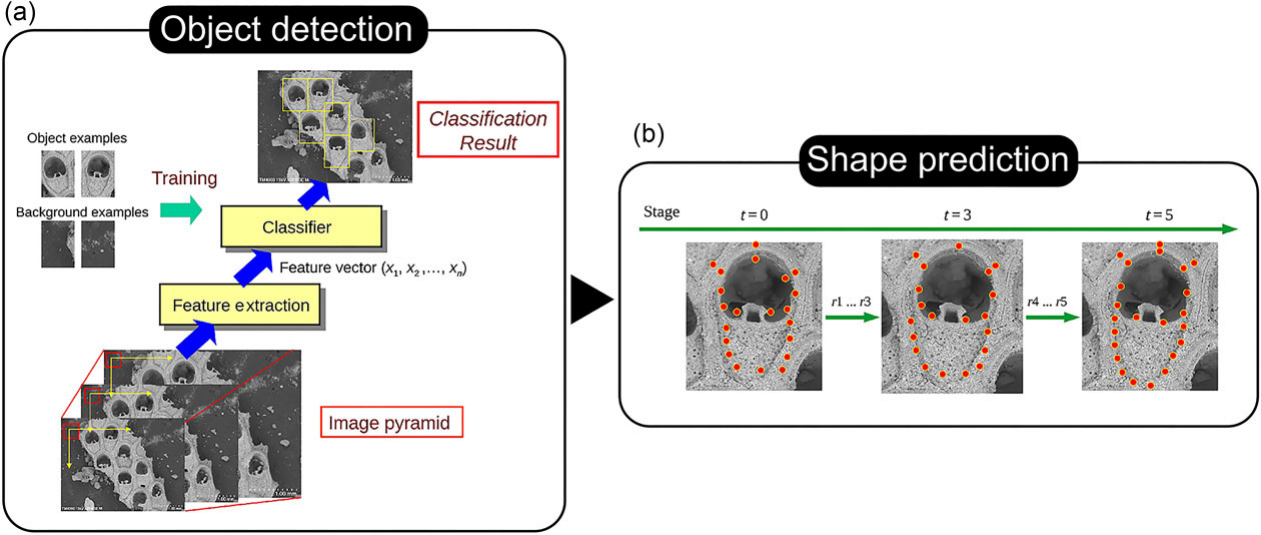
Workflow for automated landmarking in Porto and Voje (2020), showing (**a**) the object detection framework where a training set is used to first extract features and then perform classification, and (**b**) perform shape prediction using a cascade shape regression model to refine the landmark predictions. Reproduced under a Creative Commons Attribution 4.0 International License (https://creativecommons.org/licenses/by/4.0/).

AI has been successfully applied to automate placement of landmarks and semilandmarks in samples of fruit flies (Porto and Voje 2020; Salifu et al. 2022), bryozoan colonies (Porto and Voje 2020), and mice (Devine et al. 2020; Porto et al. 2021). Perhaps the most advanced implementation of DL for landmarks placement at present uses a supervised learning approach combining object detection and shape prediction to annotate landmarks (Fig. 10) (Porto and Voje 2020). Object detection, using a histogram of gradient features rather than the more common but less efficient CNN approach, was used to first identify the structure of interest, followed by shape prediction to annotate landmarks. This approach was successfully applied to three datasets of varying complexity, with object detection in particular performing well for all datasets. While only implemented for 2D images at present, the speed of data collection achieved in that study is remarkable (e.g., >13,000 bryozoan zooids annotated in 3 min, approximately the same needed to manually annotate one zooid; Porto and Voje 2020) and demonstrates the potential of AI applications to geometric morphometrics and the need to develop implementations for 3D data.

#### Landmark-free geometric morphometrics

Despite these advancements in landmark-based methods, they remain limited as they rely on homologous points of comparison. As a result, they quickly lose explanatory value with increasingly disparate taxa, as homologous points become more difficult to identify and thus fewer in number (Goswami et al. 2019). The introduction of new landmark-free approaches for the analysis of shape may allow us to overcome some of these issues, though the need for grounding in homology will always be a constraint, as well as a critical requirement for maintaining biological meaningfulness, of this approach.

Landmark-free or homology-free methods aim to describe the entire shape of specimens without using landmarks. There are several methods within this family and currently most do not directly use AI, but we note a few that are promising areas of current development. The most common approaches either decimate a mesh into a large number of pseudolandmarks (i.e., points without any homology) (Boyer et al. 2015; Pomidor et al. 2016) or use an atlas-based diffeomorphic approach (Durrleman et al. 2014; Toussaint et al. 2021). These approaches allow shapes that do not share homology to be compared and limit the loss of geometric information, but they may be sensitive to factors outside of shape, including alignment, scaling, and modality (Mulqueeney et al. 2024b). Nonetheless, they offer a potentially rich source of data for AI applications, as we discuss here with a particular emphasis on diffeomorphic methods.

Broadly, diffeomorphic methods involve a shape on a deformable grid that can be stretched and compressed, with mathematical tools called diffeomorphisms, to resemble other shapes. These methods, often referred to as methods of elastic shape analysis due to their elastic nature, can be used to quantify dissimilarities between shapes, register shapes together, and analyze morphometry, all without requiring landmarking. As described in Hartman et al. (2023), these methods can be categorized into two sections: those that apply to parameterized surfaces and those on nonparameterized surfaces (i.e., containing no known point landmarks). Techniques that incorporate these methods include large deformation diffeomorphic metric mapping (Beg et al. 2005), the square root velocity framework (Srivastava et al. 2011), and currents (Benn et al. 2019). One way elastic landmark-free techniques are proving increasingly useful is when analyzing morphometry in a 2D sense, for example, when studying the boundaries of objects seen in images. Here, instead of requiring landmarks on the boundaries, the boundary curve is analyzed as a whole (Salili-James et al. 2022a). Importantly, this also allows for possible invariances to be handled. For example, the metrics within methods can be made to be invariant to shape-preserving transformations, such as scaling, translation, rotation, and/or reparameterization (i.e., where on the boundary the curve starts/ends).

Diffeomorphic methods can also be expanded into higher dimensions as seen with open curves (Lahiri et al. 2015) and closed curves (Klassen and Srivastava 2006)—this can prove particularly useful in the analysis of curves on surfaces in evolutionary datasets. Here, elastic shape analysis allows for dimensionality reduction with a tool analogous to the classic ML tool, PCA, within the true space of the shapes of the objects in a dataset (Srivastava et al. 2011). Given these advances, there has been widespread recent research on elastic methods focused on surfaces (Jermyn et al. 2017; Pierson et al. 2021; Hartman et al. 2023).

Methods using integral geometry belong to another family of approaches used to compare the surfaces of the selected objects (Wang et al. 2021a; Lin et al. 2024). These methods can avoid issues of invariance and alignment to the same extent as the landmark-free approaches noted earlier; however, their efficacy at comparing disparate datasets currently remains untested, resulting in limited applications. Additionally, these approaches have drawn some concerns over ignoring homology (Mitteroecker and Schaefer 2022), though there is great potential for reintroducing homology by combining them with AI tools for feature or trait extraction, as described earlier and demonstrated in Case Study 5.

Overall, these approaches could be used not only to study the shape of specific homologous elements, but also could accelerate studies of modularity and integration (Zelditch and Goswami 2021), which rely on large sample sizes to assess the relationships among structures, how those relationships reflect genetic, developmental, and functional associations among traits, and how they influence the evolution of morphology over shallow to deep timescales. As with landmark-based geometric morphometrics, despite the attention being paid to new AI techniques and its great potential for automating the quantification of shape, there are at present few applications to datasets above the species level.

## Color

Color and patterning are key evolutionary components in taxa as diverse as insects, fishes, birds, and reptiles because of their importance in crypsis, aposematism, mimicry, communication, and sexual selection (Cuthill et al. 2017). Understanding how these patterns evolve is, therefore, crucial for understanding broader evolutionary themes such as natural and sexual selection, convergence, parallel evolution, and character displacement (Caro 2017). Color patterning can help researchers to recognize and discriminate between species and is commonly used in taxonomic, behavioral, and ecological studies (e.g., Sinpoo et al. 2019). Traditionally, studies have been limited to qualitative descriptions, which has restricted analyses to relatively small sample sizes due to the difficulty of manually comparing large numbers of diverse and complex patterns and color combinations (Hoyall Cuthill et al. 2024). Quantitative analyses of color patterning have become more common in recent years, with important large-scale studies being carried out in birds (Dale et al. 2015; Cooney et al. 2019) and butterflies (Van Der Bijl et al. 2020; Hoyall Cuthill et al. 2024). Automated and semi-automated methods have been developed to segment color from images (He et al. 2022; Weller et al. 2024) and to quantify and analyze color patterns (Maia et al. 2019).

ML offers a potential solution by processing vast amounts of data and using large image datasets of museum specimens for training and analysis (Case Study 7). ML uses feature extraction and classification to process images in species identification (Wäldchen and Mäder 2018), enabling the comparison of color patterning by quantifying both spectral (i.e., color and luminance) and spatial (i.e., the distribution of pattern elements) properties of color patterns across multiple specimens. This reduces the workload by removing the need to manually process images (Maia et al. 2019). One successful implementation is the analysis of field-based camera trap images, with one study that focused on Serengeti images having a 96% success rate compared with a crowdsourced team of human volunteers (Norouzzadeh et al. 2018). ML has further been used to identify individuals within species of small birds (Ferreira et al. 2020), pandas (Hou et al. 2020), and primates (Guo et al. 2020), based on only minute differences in color pattern.

The preparation and analysis of data workflows can be greatly improved with the use of AI, and some of the most significant progress in this area has been conducted on museum bird specimens. DL methods have been applied to segment and extract plumage from images, which greatly enhances the speed for processing color information (Cooney et al. 2022; He et al. 2022). This approach has also applied pose estimation methods to identify specific points of bird anatomy regions for extracting color information per body part (He et al. 2023). Automated methods are much faster and less subjective than manual methods for color segmentation but are less flexible. Van der Bijl et al. (2020) used a color profiling approach to assess sexual dimorphism in 369 species of butterflies, using a pixelated image to produce a linear sequence of coordinates containing lightness and color values. This method is effective but time-consuming because each specimen must be photographed, with images manipulated and standardized by hand, covering only 2% of the estimated 18,500 extant species of butterflies.

## Case Study 7: Color

**Fig. 11:**
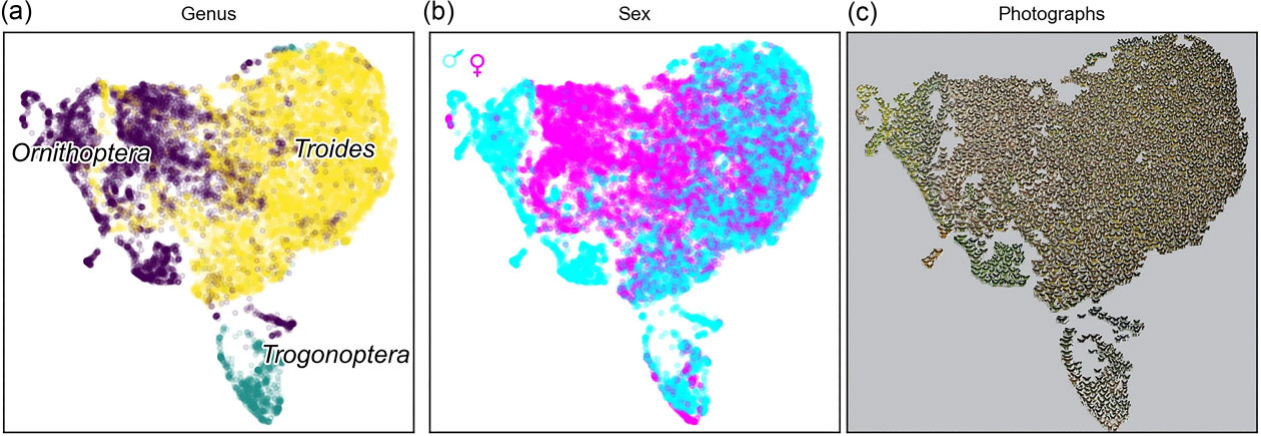
Patterns of phenotypic similarity in birdwing butterfly genera and sexes, from Hoyall Cuthill et al. 2024 and reproduced under a Creative Commons Attribution 4.0 International License (https://creativecommons.org/licenses/by/4.0/). Points represent individual photographs, and proximity represents image similarity. Shown are (**a**) genera, (**b**) sex, and (**c**) embedded images.

The wings of butterflies (Lepidoptera) are often brightly colored and conspicuous, and have evolved a high diversity of color and pattern complexity across approximately 18,500 extant species. In many species color patterns are often highly variable between sexes and are thought to have evolved through sexual selection, a hypothesis supported by behavioral studies (Panchen 1980). Qualitative observations have suggested that male birdwing butterflies (Lepidoptera: Papilionidae) can be more brightly colored than females (Vigneron et al. 2008), and that males from different regions may be visually more divergent than equivalent females. Hoyall Cuthill et al. (2024) tested these observations by using ML to quantify and characterize sexual and interspecific variation in wing patterning within this group. Euclidean spatial embeddings of 16,734 dorsal and ventral photographs of birdwing butterfly specimens (3 genera, 35 species, and 131 recognized subspecies) were generated by using DL with a triplet-trained CNN. In this method, the CNN was optimally trained to place all images so that the Euclidean distances between images from the same species are comparatively close relative to distance to images of different species (Fig. 11). CNNs are able to capture and compare features across multidimensional image embeddings and can access any variation within the image that is informative for their designated task, opening up new avenues of analysis, which were not previously possible. The approach was able to reconstruct phenotypic evolution of wing patterns and quantify sexual disparity difference for the first time, revealing high male image disparity in some species and supporting divergent selection of wing patterns in males, consistent with sexual selection. The dataset represents the entire collection of the Natural History Museum, London, the largest and most comprehensive collection of birdwing butterflies on Earth, highlighting the high-throughput ability of ML methods.

## Pose estimation

**Fig. 12:**
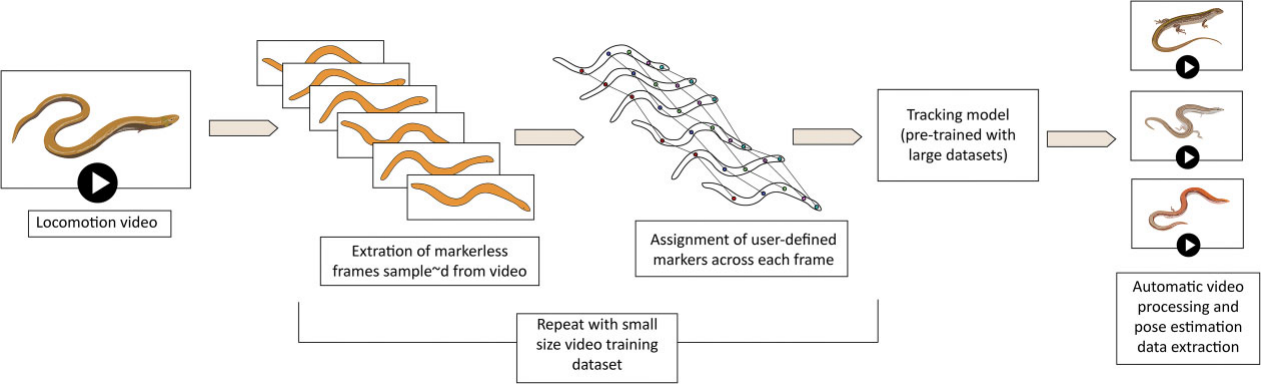
Simplified pipeline for markerless motion tracking and pose estimation from videos using DeepLabCut (Mathis et al. 2018). Limb-reduced skinks (Camaiti et al. 2023) are here used as an example of locomotion tracking.

Pose estimation predicts the relative position of body parts to each other and is used to recognize different animal poses and their changes during locomotion (Pereira et al. 2019). While estimation is usually conducted on static images (Wei et al. 2016), these capabilities have also been adapted to recognize and quantify movement (Mathis et al. 2018). Indeed, parsing kinematic patterns from videos has become the hallmark of locomotion, biomechanics, and behavioral studies, contributing to the rapid transformation of these fields (e.g., Karashchuk et al. 2021). Pose estimation is a relatively simple computer vision problem, based on the annotation of training sets from images. Originally, algorithms were unable to recognize parts that were not sufficiently distinct from the background, an issue called the “background problem” (Diaz et al. 2013), and mitigating this required the placement of markers on the moving parts prior to filming. This problem was amplified in video estimation because motion blur also constitutes a significant challenge, requiring the use of extensive and highly specific training datasets (Nath et al. 2019). In light of these issues, the main element of novelty in the field has been the development of computer vision algorithms able to handle video analyses requiring smaller datasets without markers, such as that offered by the recently introduced DeepLabCut toolbox (Mathis et al. 2018; Nath et al. 2019), which has quickly become the standard tool used for marker-free 3D pose estimation (Fig. 12). Its capabilities are based on transfer learning: the neural network it is based upon was pretrained with large datasets, allowing the application of DL to much smaller supervised datasets (Mathis et al. 2018).

Efforts are being made within the field of pose estimation to bridge gaps between biological and computer science expertise. This is increasingly important in the games and animation industries where there is a need to model animal behaviors and can be applied to evolutionary morphology. Manually editing each keyframe can be a painstaking task, and so physics-based models have been employed for years (e.g., for automatically animating horse gaits) (Huang et al. 2013). In recent years, ML tools have been incorporated to automate the process further, such as in the software, WeightShift, which combines full-body physics-based animation with AI to animate characters (Chapman et al. 2020), or in animating the locomotion of quadrupeds using neural networks (Zhang et al. 2018). Another area of pose estimation, which has recently benefited from ML, is via natural language. AmadeusGPT is a natural language interface for DeepLabCut, which integrates pose estimation and object segmentation (Kirillov et al. 2023). With this the end user can describe a query and get outputs without needing to code (Ye et al. 2023).

## Evolutionary analysis

AI has the capacity to transform our ability to capture morphology for evolutionary analysis, as detailed earlier. Thus far, it has perhaps had the greatest impact on data acquisition, which has long been a primary bottleneck for studies of evolutionary morphology. Nonetheless, we are already seeing the implementation of AI approaches for the analysis of diverse questions in evolutionary biology but these have not reached the full potential applications of AI across the field yet. Next, we discuss a range of topics within evolutionary morphology that have already benefited from AI applications and identify key areas in evolutionary morphology that are promising for development. We also provide a table of tools (Table 1) that are already available for applying AI to evolutionary morphology.

**Table 1.** Currently available tools using AI that are applicable to research in evolutionary morphology^a^

Tool name/library	Capabilities	Supported data types	Programing language	Reference
Acquiring textual data				
NLTK, spaCy *(python libraries)*	Natural language processing (NLP). For example, it can be used for extracting scientific words/taxonomic names from journal articles.	Text	Python	Bird et al. (2009)
TaxoNERD *(python library)*	Extracts scientific names, common names, and name abbreviations Can link taxa mentioned to a reference taxonomy (e.g., NCBI Taxonomy, GBIF Backbone, and TAXREF)	Tabular data, text, images	Python or R	Le Guillarme and Thuiller (2022)
Pytesseract *(python library)*	Optical character recognition (OCR) to turn images to text. Python wrapper for Google's tesseract engine.	Images	Python	Dome and Sathe (2021), Tesseract OCR (2021), and Hoffstaetter (2022)
Google Vision	Deep learning application programing interface to perform OCR	Images	N/A	Walton et al. (2020) and Vision AI (n.d.)
Deep learning				
PyTorch, TensorFlow, *(python libraries)*	DL frameworks	Tabular data (arrays, matrices, etc.) Image-based data Text Audio	Python	Abadi et al. (2015) and Paszke et al. (2019)
Scikit-learn *(python library)*	Tools for ML. Classification methods (e.g., Support Vector Machine), clustering methods (e.g., K-means clustering), dimension reduction (e.g., PCA), and neural networks.	A variety of datatypes, from tabular data to image and sound data, etc.	Python	Pedregosa et al. (2011)
PIL, scikit-image, open-cv-python *(python libraries)*	Image processing and computer vision tools. For example, thresholding, contour extraction with Snakes (Active Contour).	Images	Python	van der Walt et al. (2014)
Monai, Biomedisa *(python libraries)*	DL tools that are designed for processing medical images	Images, especially medical images	Python	Lösel et al. (2020) and Cardoso et al. (2022)
LeafMachine	DL and CV tools for trait extraction and measurement, from botanical images	Botanical images, particularly herbarium sheets	Python or GUI	Weaver and Smith (2023)
Image processing software				
ORS Dragonfly, Avizo-Amira, VGSTUDIO MAX	Softwares for processing and segmenting medical and cross-sectional images. AI-based segmentation methods are also supported.	Medical images	The software is not open-source; but it supports Python scripting	Dragonfly: Comet Technologies Canada Inc. (2022); Avizo: Thermo Fisher Scientific (2021)
3D Slicer, ImageJ, ilastik	Open-source softwares for processing medical and cross-sectional images. Users can add extensions such as SlicerMorph, and Weka Segmentation, or build new extensions.	Medical images	C++, Python, Qt	Schneider et al. (2012), Kikinis et al. (2013), Arganda-Carreras et al. (2017), Berg et al. (2019), and Rolfe et al. (2021)
Tools can be used in evolutionary morphology				
MeshCNN	Mesh classification and segmentation. Can be used for segmenting 3D mesh models of specimens.	3D mesh models	Python	Hanocka et al. (2019)
Detectron2 ML library	Object detection. Can be used for identifying a specimen in an image.	Images	Python	Wu and Kirillov (2019)
Segment Anything	A pretrained segmentation tool that can generate decent segmentation results	Images	Python	Kirillov et al. (2023)
DeepLabCut	A tool for placing keypoints on images and videos	Images and videos	Python	Mathis et al. (2018) and Nath et al. (2019)
Pl@ntNet	Species ID through identification of traits for plants	Images	N/A, input images directly to online tool (identify.plantnet.org)	Pl@ntNet IPT (2023)
FloraIncognita	Species ID and identification of traits for plants	Images	N/A, input images directly to online tool (floraincognita.com)	Mäder et al. (2021)
Fishial.ai	Species ID and feature recognition for fish	Images	N/A input images directly to web portal (portal.fishial.ai)	Fishial.ai (2019)
Merlin Bird ID	Species ID for birds from descriptions, photographs, and sound recordings	Images Audio	N/A, input images directly to mobile app (merlin.allaboutbirds.org)	Cornell Lab of Ornithology (2024)
Wolfram Mathematica	Identifying type of specimen in an image. Categorizing traits of specimens from images.	Images	Wolfram Language, C/C++, Java	Wolfram Research, Inc. (2024)
MaxEnt	Modeling ecological niches of taxa	Species occurrence data, environmental rasters	Java	Phillips et al. (2024)
Hierarchy-guided neural network (HGNN)	Combining hierarchical classification information with phenotypic data	Images	Python	Elhamod et al. (2022)

^a^We include coding libraries, websites, and software, along with their application within evolutionary morphology, the data types they support, and the programing language where applicable. The table is broken into four main sections: acquiring textual data, deep learning, image processing software, and softwares for evolutionary morphology research. This table will be regularly updated at https://phenomeai.org/.

### Clustering and classification

Classifying individual specimens is an initial step in many evolutionary studies, but is often a time-consuming task. In recent years, more efficient methods using ML image clustering have become widespread in the classification of individuals into distinct species (Punyasena et al. 2012; Barré et al. 2017; Wäldchen and Mäder 2018; Hsiang et al. 2019; Valan et al. 2019). Current research predominantly employs CNNs (Krizhevsky et al. 2017), which excel at extracting features from images and providing probability estimates to assign images to specific species classes. These methods tend to focus on classifying species and rarely describe the relationships between classes or higher-level classification, though there are some preliminary works in this area. For example, Kiel (2021) describes a method combining DL and computer vision approaches to train a CNN to categorize images of bivalve species into family groupings based on known taxonomy. For images of each species, the algorithm estimates the probability that it belongs to each family, and the results demonstrate that this approach was accurate for family-level classification and, to a lesser extent, for topology estimation.

Morphometric data are also available for use in species classification, and in recent years ML methods have been employed to accurately classify species using morphometric data (e.g., Salifu et al. 2022; Devine et al. 2023; Lin et al. 2024; Case Study 8). For instance, Elsayed et al. (2023) developed an automated approach using CNNs for identifying and classifying 2D images of tooth fossils from various animals, including sharks, elephants, hyrax, and primates. Additionally, elastic shape analysis can be used as a preprocessing technique for ML by quantifying differences between object shapes, before feeding into classic ML tools such as K-Nearest Neighbor (KNN), as in Salili-James et al. (2022a). Here, diffeomorphic methods were used to quantify differences between the shapes of objects in various 2D image datasets, from gastropod shells to leaves. A KNN classifier was then trained to classify the genus and species, based purely on the morphology of the objects in the image. Moreover, there have recently been studies that have incorporated DL techniques with elastic shape analysis, such as Hartman et al. (2021), where a Siamese neural network was trained to predict geodesic distances between curves with diffeomorphic methods to analyze and classify objects such as the boundary curves of leaves from the notable Swedish Leaf Dataset (Söderkvist 2001, 2016).

Each of these techniques must identify distinct morphological attributes for grouping, posing challenges for taxa with few specimens, such as many fossil taxa. Despite these constraints, the ability to use ML algorithms to differentiate, cluster, and classify taxa based on morphology has vast potential for fields from species delimitation and detection to phylogenetics.

## Case Study 8: Specimen classification from images

In a recent study, Hou et al. (2020) introduced the ADMorph dataset, which trained and evaluated DL models for the morphological analysis of 3D digital microfossils. The study focused on enhancing the accuracy of DL models by testing the segmentation performance of multiview CNN (Su et al. 2015), PointNet (Charles et al. 2017), and VoxNet (Maturana and Scherer 2015). The ADMorph dataset is valuable for developing and evaluating DL algorithms to precisely analyze and classify microfossil structures. Building on this foundational work, a subsequent project by Hou et al. (2021) further expands the application of DL by automating the segmentation process. This study delineated and classified approximately 500 fish microfossils within CT images, showcasing the potential of DL models to significantly streamline and enhance the accuracy of morphological analysis in paleontological research.

### Species delimitation

Species delimitation, opposed to classification, requires the ability to identify whether individuals belong to a population, which in some cases may lead to assignation of individuals as new taxonomic entities (Case Study 9). Genomic species delimitation methods have been extensively used in the last decade, including Bayesian species delimitation (Yang 2015) and unsupervised ML algorithms predicting clusters of individuals from genomic data (Derkarabetian et al. 2019). More recently, CNNs have been employed to build a morphology–molecule network that integrates morphological and molecular data for species delimitation (Yang et al. 2022). Despite their widespread adoption and increasing applications in taxonomy, these methods cannot deal with taxa that are not present in the training set, rendering them ineffective for identifying novel or undiscovered species.

Emerging techniques in one-class classification systems (Perera and Patel 2019) or open set recognition (Geng et al. 2021) offer promising avenues for extending species identification beyond initial classifications done through image analysis. However, inherent challenges remain: these techniques are currently used for outlier detection and would need to be adapted to establish species. An alternative approach would be to use phenotypic traits as a basis for delimitation. Individuals can be grouped into self-similar clusters by analyzing phenotypic traits, forming the basis for delineating populations and species (Ezard et al. 2010). Traditionally, Gaussian mixture models employing a maximum likelihood approach have commonly been used (Fraley and Raftery 2002; Baylac et al. 2003), However, the advent of deep Gaussian mixture models (Viroli and McLachlan 2019), which incorporate ML techniques, may be more suitable. These models show heightened levels of complexity, enabling them to capture intricate relationships within data. Combined with the increasing ability to acquire image or trait data rapidly, they may allow for a more nuanced and comprehensive understanding of taxonomy.

On balance, unsupervised or semi-supervised AI-based integrative taxonomic tools have the potential to play a key role in furthering species discovery. In addition to phenotypic traits, researchers are obtaining additional suites of organismal data such as acoustics, behavior, and ecology. AI will be key to bringing these complex datasets together for a biologically meaningful interpretation of a species.

## Case Study 9: Delimiting species

**Fig. 13:**
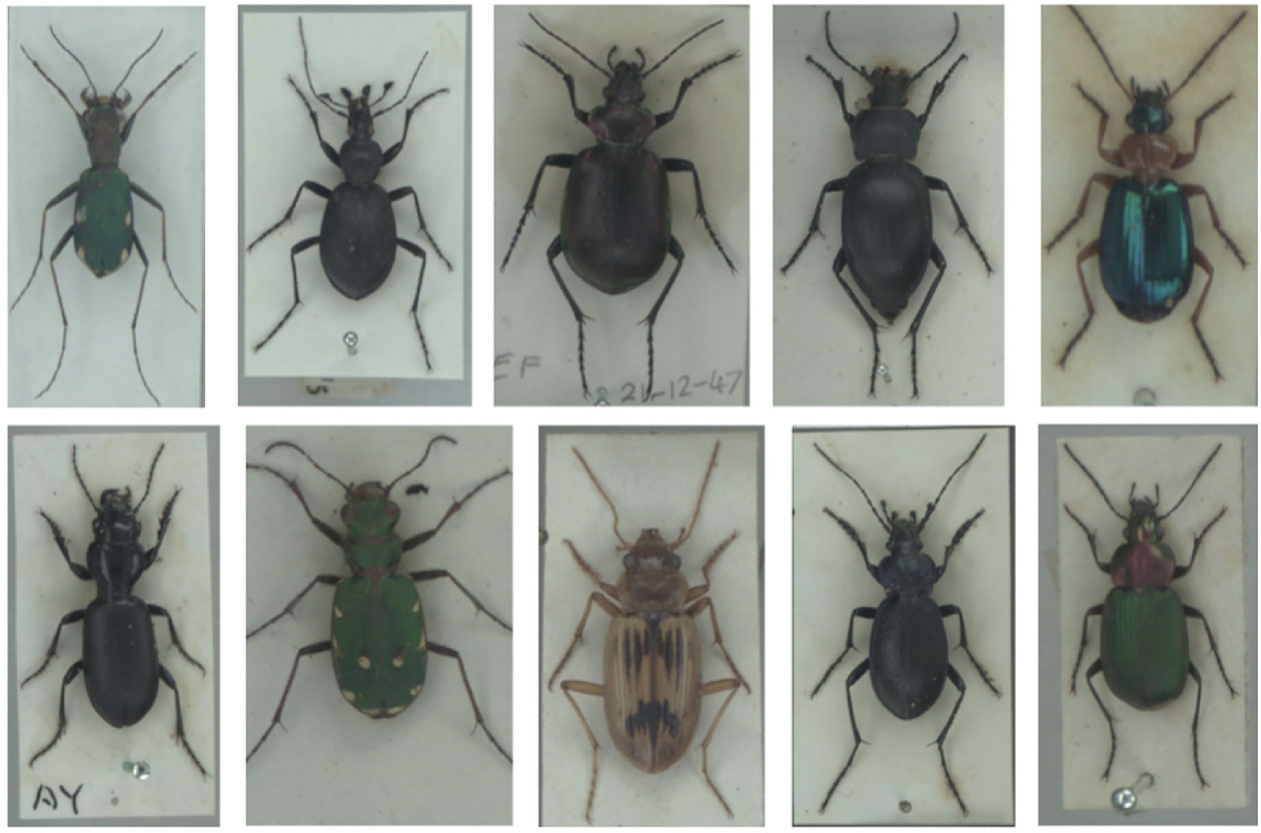
Images of carabid beetles used by Hansen et al. (2020) to train and test a CNN model for identifying beetle species. Image has been cropped from the original, and is reproduced under a Creative Commons Attribution 4.0 International License (https://creativecommons.org/licenses/by/4.0/).

Species delimitation methods have the possibility to discover new species in natural history collections. Hansen et al. (2020) created an image database of 65,841 museum specimens comprising 361 carabid beetles from Britain and Ireland (Fig. 13). A pretrained CNN model was fine-tuned on 31,533 images, validated on 25,334 images, and tested on 19,164, assigning 51.9% of test images to correct species and 74.9% to correct genus. The authors acknowledge that specimen size was a key factor in correctly identifying specimens, but model improvements may correct for this and the applications can be extended beyond high-throughput museum collections analysis to identifying species in the field using camera traps. Combined with further classification and clustering tools, such as with heatmap analysis (Hollister et al. 2023), these models can one day be used to identify potential new species.

### Phylogenies—building trees

Evolutionary studies frequently require data structured on the relatedness between taxa in the form of phylogenetic trees. ML is increasingly applied to address some of the limitations of traditional methods (Sapoval et al. 2022; Mo et al. 2024). This includes often extremely high computational expense common to Bayesian and maximum likelihood approaches (Azouri et al. 2021,^ 2023^), model misspecification (Abadi et al. 2020; Burgstaller-Muehlbacher et al. 2023), as well as issues with missing data for distance-based approaches (Bhattacharjee and Bayzid 2020). However, the accuracy and scalability of these methods remains uncertain (Sapoval et al. 2022). Additionally, a significant obstacle for ML methods is the scarcity of training data for tree inference (Sapoval et al. 2022). Due to the uncertainty associated with phylogenetic inference, a ground truth of phylogenies is fundamentally unknowable, leading to a reliance on simulated data that may not accurately reflect evolutionary relationships (Sapoval et al. 2022).

In recent decades, genetic data have dominated phylogenetic studies, due to well-studied models of nucleotide evolution and the sheer quantity of genomic data available (Misof et al. 2014; Álvarez-Carretero et al. 2022). Consequently, recent reviews of ML approaches for tree building (Sapoval et al. 2022; Mo et al. 2024) have focused on molecular-based phylogenetics instead of morphology-based phylogenetics. However, for many extinct species, molecular data are often not available, meaning morphological data must be used (Lee and Palci 2015).

Approaches that have been applied to sequence data have the potential to be adapted for use on morphological data. CNNs and RNNs have been employed to infer quartet (four taxa) topologies using simulated sequence alignments and protein data (Suvorov et al. 2020; Zou et al. 2020). Simulated quartet experiments outperform traditional methods like maximum likelihood, especially in scenarios of high substitution heterogeneities, which is challenging for many standard models (Zou et al. 2020). However, more recent analyses contest this, and traditional methods have outperformed neural network methods when the taxon number is increased above four (Zaharias et al. 2022). These methods have mostly been applied to individual sequences, but applying them to species trees involves further complexities such as incomplete lineage sorting and introgression (Maddison and Knowles 2006; Degnan and Rosenberg 2009; Suvorov et al. 2020). Restrictions of limited taxa and the complexity of species tree inference are emerging areas of research, such as in a recent study applying generative adversarial networks (GANs) to simulated data (Goodfellow et al. 2014).

While ML methods to estimate evolutionary relationships using genetic data have begun to be explored, either by approximating distances between taxa or by directly inferring topologies, application to morphological data remains challenging (Case Study 10). For molecular phylogenetics, there are many complex substitution models available to describe the evolution of sequence data (Hasegawa et al. 1985; Tavaré 1986). However, the same cannot be said for morphological evolution where the underlying processes are more difficult to model (Lee and Palci 2015) and where there is a lack of clearly defined smallest units of change across the tree of life. Methods such as Phyloformer (Nesterenko et al. 2022; Case Study 10A) still rely on models of sequence evolution to compute topologies. Without explicit models of morphological evolution or an ability to discern homology, such methods may be prone to the confounding effects of homoplasy and convergent evolution (Case Study 10B). While, even without an explicit model, phenotypic trees from ML-extracted morphological features can still closely match phylogenies based on genetic models (Hoyal Cuthill et al. 2019), the comparison between the two remains difficult.

Estimating nucleotide substitution models for large sequence datasets through traditional maximum likelihood methods is computationally intensive. More recently, DNNs were used to create ModelTeller (Abadi et al. 2020) and ModelRevelator (Burgstaller-Muehlbacher et al. 2023), two approaches to phylogenetic model selection that focus specifically on identifying the most appropriate substitution models for large datasets, which are otherwise not achievable due to computational constraints. With continuous increases in the size of datasets used for generating phylogenetic hypotheses, methods such as these will be key to assess most suitable models before phylogenies are built. While these DNNs both focus on molecular substitution models, their existence opens the possibility of developing new systems for selecting morphological evolutionary models.

One common issue for several phylogenetic methods (including maximum likelihood, Bayesian, and maximum parsimony) regardless of data type is the use of heuristic searches. In the case of Bayesian approaches, model parameters (e.g., tree topology and branch length) are adjusted and the likelihood of each adjustment is then estimated and compared. This approach explores tree space for a set number of iterations, aiming to identify more optimal parameter combinations, but it is limited by the extent of tree search, which makes it computationally expensive. Recently, ML methods have been applied to improve the efficiency of heuristic searches by predicting which neighboring trees will increase the likelihood without actually calculating the value, thereby reducing computational load (Azouri et al. 2021, 2023).

Another major challenge in both molecular and morphological phylogenetic studies is the impact of missing data, especially for distance-based methods where many of the most commonly used methods (e.g., neighbor joining) require data with no missing entries (Bhattacharjee and Bayzid 2020). In the case of molecular phylogenetic studies, this refers to missing bases in sequences. For morphological data this could be a result of incomplete specimens where certain traits or biological structures are missing or difficult to measure or score. Previous studies have shown that missing data negatively affect the accuracy of tree inference methods (Wiens 2006; Roure et al. 2013). ML methods such as PhyloMissForest (Pinheiro et al. 2022), which is a method based on Random Forest approach, and two methods proposed by Bhattacharjee and Bayzid (2020), use ML to estimate missing distance values within a distance matrix and may outperform traditional statistical methods. Overall, while there are numerous areas in which ML can improve phylogenetic inferences for diverse data types and methodological approaches, it is at present very poorly developed, particularly for morphological data.

## Case Study 10: Creating phylogenetic frameworks

(A) Molecular and ML tree building: phyloGAN and Phyloformer

**Fig. 14:**
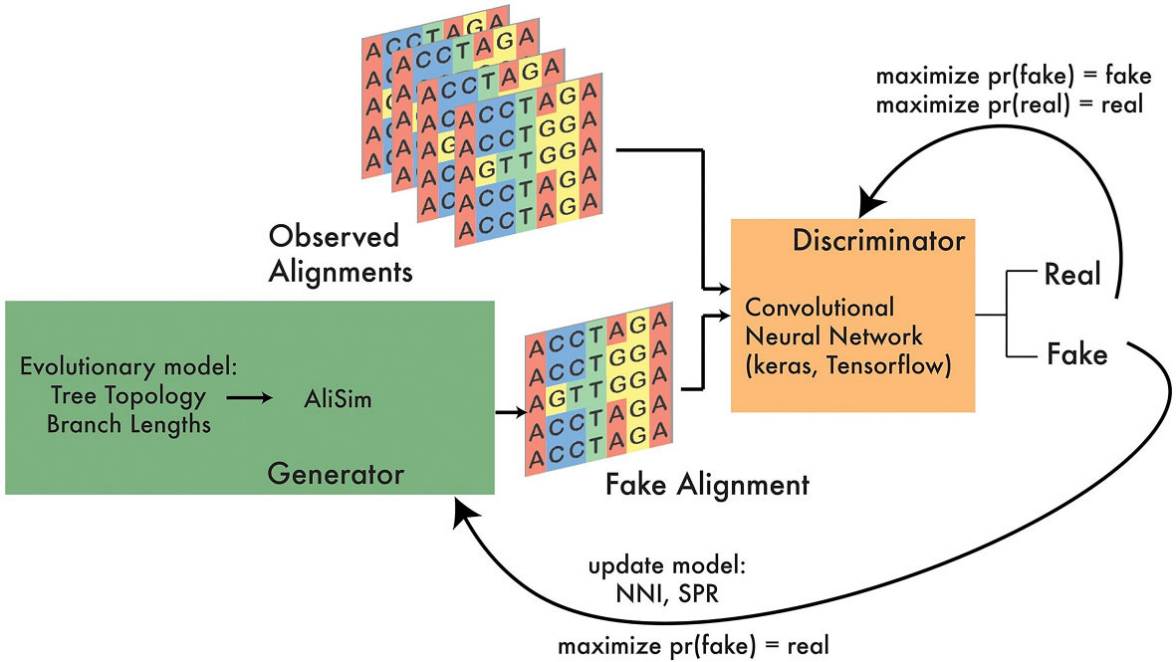
Overview of phyloGAN (Smith and Hahn 2023) wherein a generator generates tree topologies with branch lengths utilizing nearest neighbor interchange (NNI) and subtree pruning and regrafting (SPR) methods. These trees are then evaluated within the discriminator to identify real versus generated data. Reproduced under a Creative Commons Attribution 4.0 International License (https://creativecommons.org/licenses/by/4.0/).

ML-based phylogenetic inferences have been limited to inferring unrooted tree topologies for quartets of taxa as the number of plausible trees increases exponentially with increase in the number of taxa (Felsenstein 1978; Suvorov et al. 2020). The phyloGAN model is an ML approach that utilizes heuristic search strategies through GAN to build trees with molecular data (Smith and Hahn 2023). It uses two networks: a generator that suggests new topologies, and a discriminator trained to differentiate real and generated data, estimating the fit of proposed topologies and alignments (Fig. 14). This method imitates the heuristic search employed by many traditional methods to explore tree space for more optimal trees. PhyloGAN shows an improvement in the number of taxa that can be considered compared to previously mentioned methods as demonstrated using seven species of fungi, but is still limited compared to traditional methods, and hampered by lengthy computational times (Smith and Hahn 2023).

Another molecular ML tree building approach is Phyloformer, which computes distances between molecular sequences in a multiple sequence alignment (MSA) (Nesterenko et al. 2022). This method simulates trees, and then uses probabilistic models of sequence evolution, working backward to simulate MSAs. Supervised learning is used to train a transformer-based model to reverse-engineer the phylogeny based on an associated MSA. For Phyloformer, the algorithm estimates pairs of evolutionary distances between sequences that can then be used to infer a tree, using traditional methods such as neighbor joining. Phyloformer outperforms standard distance-based methods, as well as maximum likelihood due to its higher computational speed.

(B) Convergence in morphological ML tree building

One of the key challenges in utilizing morphological data to build phylogenetic trees has been morphological convergence. Kiel (2021) used feature extraction to estimate family-level classifications of bivalves and by grouping them into orders and subclasses, has attempted to overcome the limitation posed by convergence of traits. They then used the degree of certainty in clustering as a proxy for morphological similarity between families of bivalves. These probability scores are used as a proxy for morphological similarity and to construct a distance matrix, which was in turn used to cluster the families and infer a topology. While this method did find significantly more bivalve families clustering with members of their known subclasses than expected by chance, the resulting phylogeny did indicate many unlikely placements. When multiple CNNs trained at different taxonomic levels were combined, the resulting phylogeny more closely matched the expected clustering based on existing taxonomic standing.

(C) Supplementing morphological data with “known” phylogenetic data

**Fig. 15:**
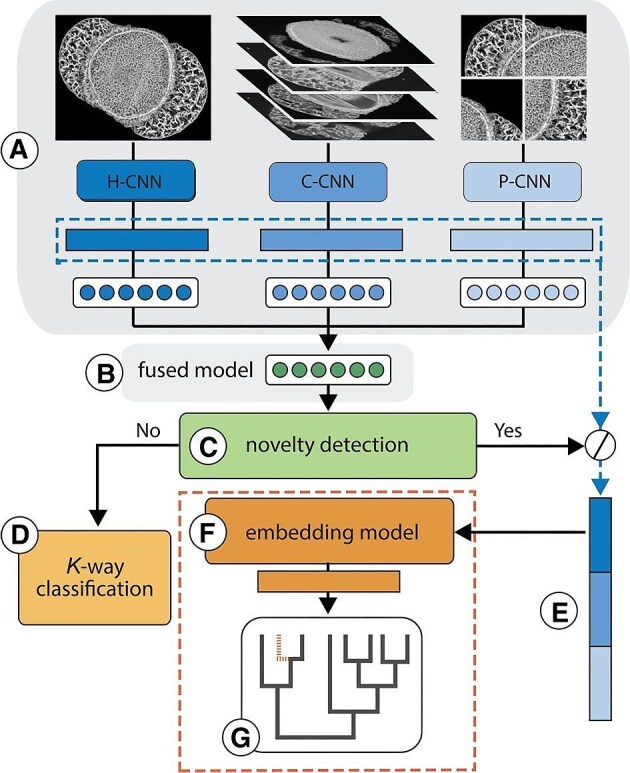
Three neural networks trained in Adaïmé et al. (2024) to score the shape, internal structures, and texture. The scores are combined and images are classified into known versus new taxa. Reproduced under a Creative Commons CC-BY-NC-ND license.

Adaïmé et al. (2024) present a novel method incorporating preexisting phylogenetic information into ML training. Pollen grains were classified into “known” or novel taxa by multiple CNNs trained on morphological characters (shape, internal structure, and texture). The morphological features of these taxa were then fed into a phylogenetic embedding model, which uses a preexisting molecular phylogeny as the ground truth. The model uses “known” phylogenetic positions of taxa as a template to guide how the morphological characters are used to estimate phylogeny, and is considered accurate if it can use morphological characters of known taxa to infer a phylogeny closely matching the molecular phylogeny, and subsequently to place novel and fossil taxa (where genetic data have not or cannot be obtained) into the topology (Fig. 15). They tested the accuracy of the method by taking taxa that had “known” phylogenetic placements and treating them as if they were novel. The model placed these pseudonovel taxa in their “correct” respective subclades with high support. By transforming morphological characters based on preexisting phylogenetic information, this method improves upon clustering based on morphological similarity alone. However, there are potential concerns over the assumption of a ground truth in phylogenetics.

### Phylogenetic comparative methods and evolutionary modeling

Using a phylogenetic framework to estimate the evolution of clades and traits has become a core part of evolutionary morphology over the past few decades (Felsenstein 1985; Adams and Collyer 2019). Analysis of trait variation across phylogenies and through time relies on the availability of well-supported topologies and time calibration. Recent advances in genome sequencing and big data approaches to taxonomic sampling and trait data collection have increased the availability of time-calibrated phylogenies (Álvarez-Carretero et al. 2022). In turn, this has enhanced our ability to reliably map the evolution of traits on phylogenies and consider phylogenetic relations when examining relationships between traits across multiple taxa.

The potential of AI to reconstruct trait evolution within a phylogenetic framework has been theoretically documented. For instance, Ruder (2017) described a multitask learning approach that involves an ML framework consolidating data from various tasks. It is achieved through an algorithm that minimizes the variance of estimators by employing a penalty term to align models more closely, facilitating the simultaneous estimation of ancestral states for multiple characters. Similarly, Ho et al. (2019) illustrated the theoretical application of ML to the ancestral estimation of phenotypic traits through a multitask learning approach applied to Brownian motion models of continuous biological traits. The study showed that this approach enhanced ancestral estimations compared to maximum likelihood models, albeit with a minor bias introduced in the phylogenetic estimates.

Despite theoretical advances, there are currently few practical applications of ML approaches to estimate trait evolution. A known issue that would benefit from an AI-based modeling approach is the assignment of distinct rates of character evolution to different parts of a given phylogenetic tree (i.e., King and Lee 2015). ML would enable the simultaneous pooling of multiple data sources, including distributions of states at the tips of phylogenetic trees, branch lengths, node ages, uncertainty in node resolution, and hidden states, and consideration of a wide variety of complex models that may better reflect phenomic datasets (Goswami and Clavel 2024). This would allow the assessment of trait covariations, studies of modularity and integration, changes through time using existing phylogenies, and probabilities of key innovations versus gradual variations.

ML approaches could also facilitate the comparison of simulations across trees. Furthermore, ML methods could account for phylogenetic relatedness in analyses of trait correlations. In the field of bioinformatics, using DNN and convolutional graph network (CGN) architectures in phylogenetic profiling for protein interactions improved predictions (Moi and Dessimoz 2022). In particular, combining CGN with a graphical representation of tree topology allowed for prediction across multiple species and could be used to predict pairwise interaction across time. Using these algorithms in conjunction with phylogenetic information is currently exploratory but could potentially streamline and improve multiple aspects of estimating trait evolution and ancestral states, allowing better modeling of the complex factors underlying evolution on a phenomic scale (Niemi 2020).

### Function and adaptive landscapes

In evolutionary biology, adaptive landscapes are conceptual frameworks that illustrate the relationship between the phenotype of an organism and its fitness within a specific ecological context (McGhee 1980; Simpson 1984; McGhee 1999). They provide a visual representation of natural selection-driven trait space across the blanket of an adaptive landscape, where peaks of specific traits reflect higher fitness compared to putative trait space across the landscape (Arnold 2003). Over evolutionary time, genetic variation, mutation, recombination, and natural selection drive the population toward regions of higher fitness (Arnold et al. 2001). Utilizing models of trait diversification can be helpful in tracing adaptive peaks of species through time, adapting to different ecological niches or responding to environmental shifts (e.g., Martin and Wainwright 2013). The study of adaptive landscapes is key both to understanding the evolutionary adaptive mechanisms giving rise to biodiversity and predicting the future adaptive potential of species in light of anthropogenic-driven habitat loss and climate change.

Functional adaptive landscape analysis uses the morphology and function of skeletal elements to model landscapes (Polly et al. 2016; Dickson and Pierce 2019; Jones et al. 2021; Tseng et al. 2023). In paleontology, functional adaptive landscapes commonly employ finite element analysis (FEA) as a functional metric (Polly et al. 2016; Deakin et al. 2022). ML algorithms can replace FEA to predict the behavior of a beam in a one-dimensional system if the algorithms are first trained on initial FEA. Neural networks have been suggested to provide more accurate FEA results than boosting regression trees or random forest algorithms (Vurtur Badarinath et al. 2021). Additionally, AI has been increasingly applied to FEA-based biomechanical modeling (Liu 2019; Galbusera et al. 2020; Mouloodi et al. 2021). These techniques can be applied to data extracted from static images, 3D image data (Galbusera et al. 2020), and even motion capture (Mouloodi et al. 2021). This is particularly useful for the creation of models of the range of appendicular motion, relationships between internal organs, and even models of cytokinesis (Huiskes and Hollister 1993; Ross 2005; Shi et al. 2010).

### Phenome–environment and ecometrics

One of the most established areas of phenotypic analysis is quantification of relationships between phenomes (the sum of phenotypic traits) and the environmental context in which they evolved (e.g., DeGusta and Vrba 2005; Stubbs and Benton 2016; Panciroli et al. 2017; Benevento et al. 2019). The end goal of many studies using this approach is to assign an ecomorphological characterization to phenotypic traits and to parse their ecological signal (e.g., Fortuny et al. 2011; Barr 2018). AI has been implemented in this field through the use of algorithms that infer present and past ecomorphologies by reducing the dimensionality of ecomorphological data through methods such as random forest (Spradley et al. 2019; Rabinovich 2021; Sosiak and Barden 2021; Mahendiran et al. 2022). Similarly, ML procedures have been used to discriminate and sort phenotypes based on their belonging to specific ecomorphs or ecological guilds (MacLeod et al. 2022). These studies have highlighted the advantages of AI-based approaches compared to standard procedures used to test the links between morphology and ecology, such as canonical variate analysis (Albrecht 1980).

The related field of ecometrics is a taxon-free approach to quantifying the distribution of functional traits across space and time (Eronen et al. 2010). Ecometric correspondence between environmental and phenotypic data is used to develop transfer functions, which can be used to reconstruct paleoenvironments or incorporate species distribution modeling (SDM) to model future spatial distributions of phenotypes given predicted climatic scenarios (Vermillion et al. 2018; Parker et al. 2023). Existing work uses linear and maximum likelihood approaches to perform ecometric modeling. These approaches have a limit of one or two climate inputs, normally limiting analyses to consider only annual precipitation and mean annual temperature (Parker et al. 2023). However, using a random forest approach would enable the model to use any number of climatic variables. Similarly, SDMs can be built using CNNs, capturing nonlinear transformations across multiple variables (Botella et al. 2018). DL approaches to quantifying phenome–environment would enable models to better approach the complex factors contributing to climate and trait distribution, as in studies of trait evolution.

### Niches and niche evolution

ML algorithms, including boosted regression tree and random forest, have become standard methodologies for modeling the ecological niches of taxa and, by extension, their potential spatial distribution. Over the past decade, research has extensively focused on predicting the ecological effects of climate change by using ecological niche modeling (Qin et al. 2017; Deb et al. 2020; Tang et al. 2021; Karuppaiah et al. 2023). The most prominent ML model in this area is the maximum entropy modeling method (MaxEnt), which has been applied in thousands of studies since its introduction (Phillips et al. 2006; Merow et al. 2013).

MaxEnt's ubiquity in scientific literature is in part due to the algorithm requiring relatively few inputs (only species occurrences and geographic data) and relying on biologically reasonable assumptions. It assumes that a taxon will occupy as large an area as possible (maximum distribution entropy; Phillips et al. 2006; Elith et al. 2011). These limitations have also produced an abundance of literature critiquing and subsequently optimizing MaxEnt's statistical assumptions and processes (Cobos et al. 2019; Low et al. 2021; Sillero and Barbosa 2021; Campos et al. 2023).

Studies that use MaxEnt or other ML methods tend to consider niches as static entities, with many publications “projecting” the same niche onto environmental rasters representing distinct points in time, sometimes thousands or millions of years ago (Saupe et al. 2019). Niche evolution studies have instead relied on measuring the contemporary niche overlap of different taxa (usually via the methodology of Broennimann et al. 2012), considering the similarities and differences within a phylogenetic context (Doré et al. 2023; Padilla-García et al. 2023; Vasconcelos et al. 2023). While both approaches are useful in understanding ecological evolution across time, they are limited by their discrete temporal sampling—niches change continuously across space and time, and an individual niche of a taxon may also change over time.

ML methods could be developed further to identify and accommodate niches changing over time. Taxon occurrences sometimes have associated temporal metadata, which could be used by an AI tool to predict the continuous changes in a niche in the recent past or near future. This could prove especially invaluable in studying the effects of climate change at a higher resolution. Considering a geological timescale, the predicted ecological niches of fossil taxa (modeled with environmental data representing periods in deep time) could be used to calibrate and, thus, further validate continuous niche evolution models across phylogenetic trees.

## Prospectus

The scope of evolutionary biology is immense, involving the history of life on Earth over the past 3.7 billion years. For the vast majority of species that ever lived, the only available data are morphological in nature; thus, studying morphology is crucial for understanding the evolution of organisms. Yet, methods for capturing morphological data remain largely manual, presenting a bottleneck for the study of morphological evolution, particularly in comparison to other biological fields with mature methods for “omics”-level analyses. The use of AI is bringing about a massive transformation in the field of evolutionary morphology, both for data capture and analysis. Integrating AI techniques into this area will become increasingly important as the field continues to move toward larger-scale analyses and bigger data.

As we have discussed, AI has been successfully applied to a range of data acquisition for evolutionary morphology, and is only increasing in the pace of development and accessibility for nonexperts. For example, AI is already making it quicker to generate, refine, and access image data of larger quantities and/or greater resolutions than ever before. Large gaps remain, however, including discriminating features or ROIs, extracting discrete traits or 3D morphometric data in datasets with large amounts of variation (which are common in comparative evolutionary analysis), and in applying AI for improving evolutionary models for morphological data. There are also numerous challenges with making AI tools accessible to nonspecialists and finding affordable and sustainable solutions for the storage, annotation, and processing of datasets that are larger in both number and size. These areas should be the focus of efforts over the coming years. While we have detailed applications of AI to several research areas in morphological evolution, there are many more for which AI has yet to make a significant impact. Next, we note a few subfields of evolutionary morphology that have clear pathways for improvement through AI. Finally, we close with some considerations on the accessibility and environmental effects of using AI in research.

### Emerging fields

#### Retrodeformation

Several studies have demonstrated that fossil data are critical for accurately estimating phenotypic evolution through deep time (e.g., Slater et al. 2012; Goswami and Clavel 2024, and references therein). A common challenge in paleontology is encountering fossils that have undergone taphonomic distortion via brittle or plastic deformation (Schlager et al. 2018; Kammerer et al. 2020). This distortion can severely hinder attempts to assess and quantify intra- and interspecific shape by introducing nonbiological variation. Consequently, in addition to the lack of integration in phylogenetic analyses, fossil data are often excluded from geometric morphometrics and phylogenetic comparative methods. Retrodeformation is the process of restoring the original shape of an object by reversing taphonomic distortion (Lautenschlager 2016; Herbst et al. 2022). While landmark- and symmetry-based procedures to manually perform these operations are available (Schlager et al. 2018), they are time-consuming and can only be applied to relatively small datasets, limiting the taxonomic breadth of studies.

AI provides an opportunity to automate and enhance this process. ML models, such as neural networks, can be trained to recognize and correct specific types of deformations. These models can learn patterns of distortion and apply appropriate corrections. In the future, AI may aid in the reconstruction of 3D objects or scans of distorted or even completely flattened fossils, thereby helping to recover valuable 3D morphology. Once models have been trained on a dataset of naturally distorted fossils and manually performed retrodeformation simulations, they can be integrated into software applications or embedded in hardware systems for real-time correction and analysis. The choice of AI algorithms will depend on the specific application and the nature of the deformations to be corrected. For instance, de Oliveira Coelho (2015) used logistic model trees to predict the temperature at which human bone was burnt. Similarly, Zeng et al. (2021) used a support vector machine algorithm to detect small geological faults. Such methods could be adapted to estimate the extent of brittle and ductile deformation a fossil has undergone, thereby enabling evolutionary morphologists to apply the opposite forces to correct the distortion.

#### Histology

Histology examines the microscopic structure and morphology of tissues, encompassing both contemporary and fossil tissues in the field of paleohistology. Historically, paleohistology has provided insights into growth, physiology, and development, while its application has expanded to investigate tissue form and function. For instance, Bailleul et al. (2012, 2019) explored the function of duck-billed dinosaur dental batteries through paleohistological techniques. The advent of AI tools has significantly advanced histology, particularly in histopathology, enhancing cancer recognition and clinical oncology (Shmatko et al. 2022). AI holds promise for increasing throughput in pattern recognition tasks. Current applications of AI in biological research include the quality assessment of histological images (Haghighat et al. 2022) and the characterization of herbivore diets through microhistological analysis (Filella et al. 2023). Moreover, neural networks have been employed to identify primary and secondary osteon regions, producing segmented maps of various osteon regions. This segmentation, in conjunction with phylogenetic analysis, has elucidated developmental pathways leading to miniaturization in theropod dinosaurs (Qin et al. 2022b). The potential for AI in histological studies is substantial, particularly within the context of investigating evolutionary morphology using landmark-free morphometrics, marking it as a promising avenue for future research.

#### Genome–phenome mapping

AI has been applied in two main areas of genome–phenome association (GPA): the medical sciences and food production. This is not surprising, as both are umbrella areas of research with high societal impact. Different AI algorithms have been applied in a variety of genome-wide association studies related to human health, helping to link genetic variants to different pathologies in complex ways (Long et al. 2023). Neural network approaches have also been developed to both understand the association between small genomic mutations and clinical phenotypes (Mieth et al. 2021) and also the complex correlation between microbial communities and diseases (Liu et al. 2021a). In an agricultural setting, similar neural network approaches have been used to predict potential phenotypes in genetically modified rice crops (Islam et al. 2023). More complex approaches have recently been tested on both human and agricultural datasets and have been found to predict not just the genomic or phonemic component, but also potential new associations between the two through the use of weighted deep matrix GPA (Tan et al. 2022). GPA approaches have clear implications for the future of evolutionary biology and phenomics studies, for example by allowing to connect morphological changes with specific mutations. Although these methods are still in their infancy and have yet to find wider applications outside of the medical and agricultural fields.

#### Evo-devo

ML has been successfully applied to the study of gene expression in embryonic development of model organisms (Feltes et al. 2018; Naert et al. 2021; Čapek et al. 2023). Algorithms have also been developed to aid in phenotyping and staging embryos and to recognize diseases and malformations (e.g., Jeanray et al. 2015; Al-Saaidah et al. 2017). In evolutionary developmental biology (evo-devo), these methods have only recently been applied to phenotype identification. A few pilot studies have been conducted using both image and morphometric data on human cells, model organisms, and plants (Masaeli et al. 2016; Cai and Ge 2017; Chen et al. 2020). CNNs have been used to extract visual patterns from images, to aid embryo staging, and to analyze changes in phenotype during ontogeny (Feltes et al. 2018; Naert et al. 2021). Through further development of these methods and their applications to nonmodel organisms, it will be possible to conduct more through studies comparing developmental phenotypes across multiple lineages.

#### Data engineering

Focus should be not only on potential fields and AI methods but also on the morphological data itself. Data engineering involves the preparation of data before any analysis or methods can be applied—this is undoubtedly a crucial aspect of AI as a whole. With the acceleration of data acquisition, along with previously collected data (MorphoSource: Boyer et al. 2016; Phenome10K: Goswami 2015; DigiMorph: Rowe 2002), the volume of usable data is increasing. In order to take full advantage of the potential of AI and “big data” in evolutionary morphology studies, first the previously collected data should be transformed into AI-ready formats. This data is then suitable to be used to explore learning strategies such as self-supervised learning. Morphological data, similar to medical data, often require extensive domain knowledge for labeling, leading to the time-consuming creation of labeled training sets. Therefore, using unlabeled data in training could be a viable option. To have better data and performance evaluation, interdisciplinary collaboration is essential; biologists can help AI experts in tailoring methods to better suit the specific data. Preparing these large phenomics datasets for algorithm training is the first step to integrate AI methods into large-scale phenomics studies.

### Accessibility and considerations

Until very recently, most AI models were built and applied using Python libraries such as Caffe, TensorFlow, and PyTorch (Jia et al. 2014; Abadi et al. 2015; Paszke et al. 2019), requiring both AI and programing knowledge. Additionally, running these models required specialized, expensive hardware, such as GPUs, which are commonly used in training AI models. Consequently, the required level of expert understanding of AI and costly hardware restricted the accessibility for many researchers in the biological sciences.

As AI continues to advance, it is becoming increasingly accessible to nonexperts and more affordable to implement due to several factors: (1) increasingly user-friendly software has reduced the need for in-depth AI-related knowledge; (2) the growth of open-source and pretrained models has significantly reduced the computational resources, data, and time required to develop AI models; and (3) the advent of cloud-based AI services has allowed researchers to access powerful AI models without investing in local GPUs. Furthermore, with the expansion of large language models in the public domain, via services such as ChatGPT (Brown et al. 2020), many researchers can now use AI to learn how to code, enabling them to program and train their own models (Cooper et al. 2024).

Despite these advancements, there are many challenges and certain aspects that require a degree of caution. AI relies on the data it has been trained on, and the people who have developed it. Biases in data, as well as cognitive bias, can cause or emphasize biases in the algorithm, and can lead to incorrect results (Mehrabi et al. 2021; Zhang et al. 2022). Attention must also be given to data cleaning and preprocessing to mitigate unrefined training data. Additionally, when it comes to data within the natural sciences, especially within the public domain, particular attention must be given to maintain consistency during the data processing stages, for example, to ensure morphological or taxonomic annotations are following standard framework.

Moreover, even with cloud-based AI services, storage and processing power are expensive and will be limiting factors for many researchers. Additionally, the environmental impact of AI cannot be overlooked, particularly as many studies in our fields aim to protect the natural world and limit human-caused climate change and destruction of biodiversity. Evolutionary morphology studies increasingly involve the collection and storage of large quantities of image data. These datasets are currently limited by the hours of manual input required, but will only increase in size as AI approaches allow for more efficient processing and analysis, leading to larger, more complex studies that in turn require increased hardware and energy input. Training large-scale models can consume substantial amounts of energy, contributing to carbon emissions, although admittedly the models trained and used in evolutionary biology are unlikely to be as large as those from tech giants like Google, Meta, and OpenAI. Some studies using large-scale genetic datasets have estimated the carbon footprint of their computational analyses (Philippe et al. 2019; Qin et al. 2022a). More formal approaches to sustainable computer science are being developed in the form of emission calculation tools (Lacoste et al. 2019; Lannelongue et al. 2021), assessments of their suitability for various approaches (Bouza et al. 2023), and proposed principles for greener computational science in the future (Lannelongue et al. 2023). As the scale of AI models and the demand for AI continue to grow, it will be increasingly important for us to evaluate the environmental impact of future studies in evolutionary morphology.

To conclude, we have here provided an introduction and overview of the current and potential future applications of AI to evolutionary morphology. At present, many of these methods remain technical and difficult to apply, due to the need for advanced coding knowledge and access to good hardware such as high-memory GPUs or high-performance computing systems. Developments are, therefore, required to make these methods more widely accessible and to allow for greater understanding and addressing of their capabilities and limitations. As AI becomes more accessible and tailored toward applications central to the study of evolutionary biology, we expect that it will transform the study of evolutionary morphology. By accelerating and improving capture and analysis of “big data” on phenotype for diverse comparative datasets, AI will allow the realization of evolutionary phenomics and launch a new phase in the study of past and present biodiversity.

## Data Availability

No new data were generated or analyzed in support of this research. The tools table in this paper will be kept updated at http://www.phenomeai.org/.
